# Authentication of *Garcinia* fruits and food supplements using DNA barcoding and NMR spectroscopy

**DOI:** 10.1038/s41598-018-28635-z

**Published:** 2018-07-12

**Authors:** Gopalakrishnan Saroja Seethapathy, Margey Tadesse, Santhosh Kumar J. Urumarudappa, Srikanth V. Gunaga, Ramesh Vasudeva, Karl Egil Malterud, Ramanan Uma Shaanker, Hugo J. de Boer, Gudasalamani Ravikanth, Helle Wangensteen

**Affiliations:** 1 Department of Pharmaceutical Chemistry, School of Pharmacy, University of Oslo, P.O. Box 1068, Blindern, 0316 Oslo Norway; 2 Ashoka Trust for Research in Ecology and the Environment (ATREE), Royal Enclave, Srirampura, Jakkur Post, Bangalore, 560064 India; 3 Natural History Museum, University of Oslo, P.O. Box 1172, 0318 Oslo, Norway; 4 Department of Crop Physiology, School of Ecology and Conservation, University of Agricultural Sciences, Gandhi Krishi Vigyan Kendra, Bangalore, 560065 India; 5 Department of Forest Biology, College of Forestry, University of Agricultural Sciences, Sirsi, 581401 India

## Abstract

*Garcinia* L. (Clusiaceae) fruits are a rich source of (−)-hydroxycitric acid, and this has gained considerable attention as an anti-obesity agent and a popular weight loss food supplement. In this study, we assessed adulteration of morphologically similar samples of *Garcinia* using DNA barcoding, and used NMR to quantify the content of (−)-hydroxycitric acid and (−)-hydroxycitric acid lactone in raw herbal drugs and *Garcinia* food supplements. DNA barcoding revealed that mostly *G*. *gummi-gutta* (previously known as *G*. *cambogia*) and *G*. *indica* were traded in Indian herbal markets, and there was no adulteration. The content of (−)-hydroxycitric acid and (−)-hydroxycitric acid lactone in the two species varied from 1.7% to 16.3%, and 3.5% to 20.7% respectively. Analysis of ten *Garcinia* food supplements revealed a large variation in the content of (−)-hydroxycitric acid, from 29 mg (4.6%) to 289 mg (50.6%) content per capsule or tablet. Only one product contained quantifiable amounts of (−)-hydroxycitric acid lactone. Furthermore the study demonstrates that DNA barcoding and NMR could be effectively used as a regulatory tool to authenticate *Garcinia* fruit rinds and food supplements.

## Introduction

Globalization in the trade of herbal products and an expanding commodity market have resulted in widespread consumption of medicinal plants as drugs, cosmetics and food supplements, both in developing and developed countries^1,2^. Quality, safety and efficacy of herbal medicines are key requirements for public health and a major concern for regulatory authorities^3^. In India, herbal raw materials for the herbal industry are predominately harvested from wild populations^4^ .Unsustainable harvest of medicinal plants from the wild can lead to severe ecological consequences, including reduced plant populations, habitat destruction, loss of genetic diversity, and local extinction of species^5^. India is the second largest exporter of medicinal plants and exports mainly in the form of dried plant products^4^. Several reports suggest that only 10% of medicinal plants traded in India are being cultivated^2,4^. Growing commercial demands for raw drug products increases the incentive for adulteration and substitution in the medicinal plants trade, and such adulteration can threaten consumer health, damage consumer confidence, and generally lower the trade value of such products^1^.

**Figure 1:**
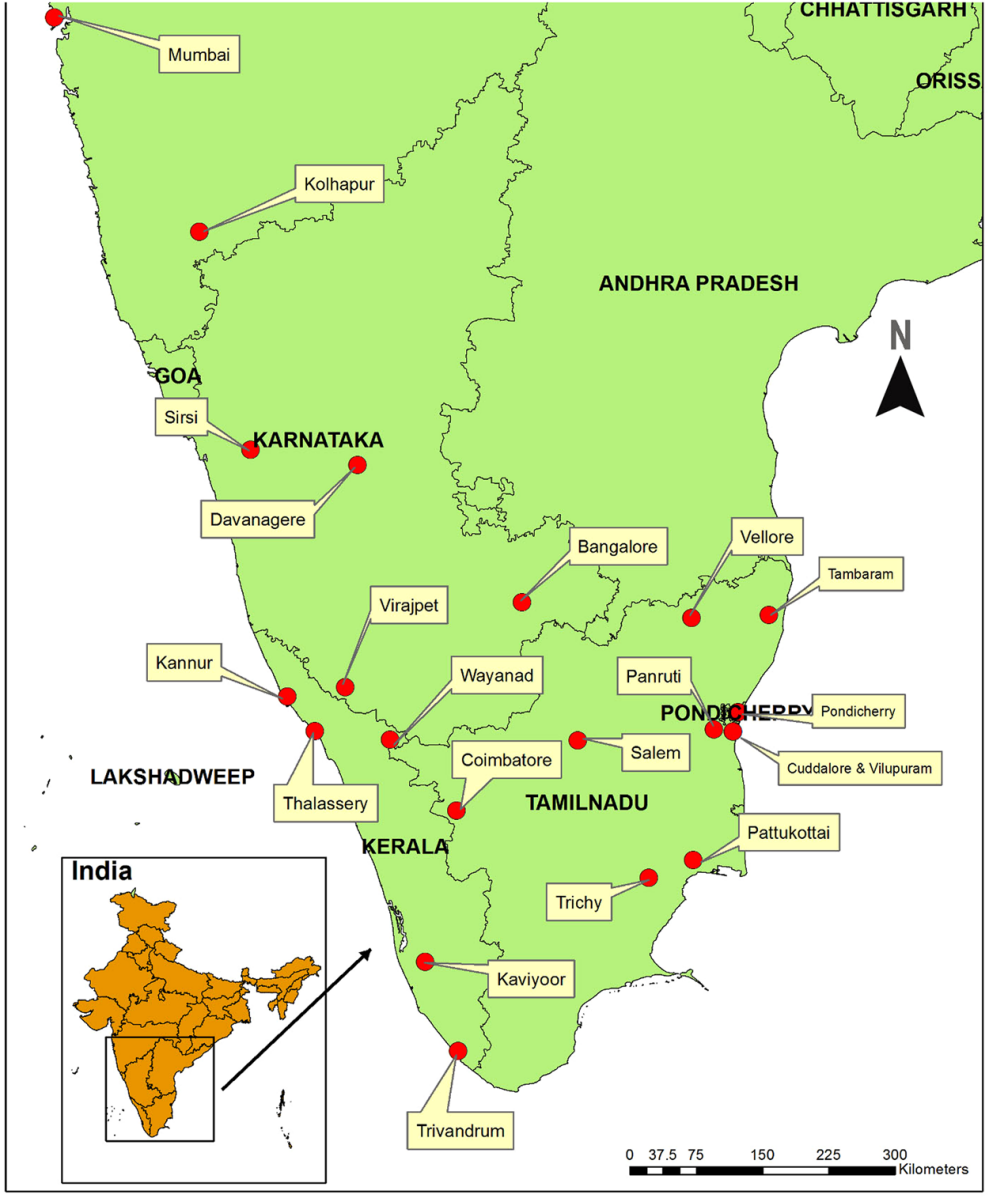
Places of collections of *Garcinia* raw drug samples in South India.

*Garcinia* L. (Clusiaceae) is a genus of evergreen polygamous trees and shrubs comprising 400 species with a pantropical distribution^6^. Many *Garcinia* species in tropical Asia, Southern Africa and Northern Australia are locally used, and the edible fruits are of interest worldwide, as well as having potential implication on the economy of local communities^7^. Thirty-five species occur in India, of which 17 species are reported from the Western Ghats^7^. *Garcinia* fruits are widely collected and commercially exploited for their medicinal value, and the fruit rinds of *Garcinia gummi-gutta* (L.) Roxb. (syn. *Garcinia cambogia* (Gaertn.) Desr.) are traditionally used to treat constipation, piles, rheumatism, edema, irregular menstruation and intestinal parasites, and are also used as a food flavoring agent and preservative^8^. The fruit rinds of *Garcinia indica* (Thouars) Choisy are traditionally used to treat rheumatic pains, bowel complaints, hemorrhoids, ulcers, inflammations, sores, dermatitis, dysentery, to prevent hyperhidrosis, and are an important culinary agent, as well^9^. In India, *G*. *gummi-gutta* and *G*. *indica* are the predominant *Garcinia* species occurring in the Western Ghats, and these are locally traded as *Kodampuli* and *Kokum*, respectively (Fig. 1). These species are traded throughout India and exported to other countries as raw drugs, juices and extracts^7^. Several other species, such as *G*. *morella* (Gaertn.) Desr., *G*. *cowa* Roxb. ex Choisy, *G*. *mangostana* L., *G*. *kydia* Roxb., *G*. *lanceifolia* Roxb. and *G*. *pedunculata* Roxb. ex Buch.-Ham, are also traditionally used and traded for various culinary and medicinal purposes^7^. The trade analysis of *Garcinia* species suggests that the demand for the dried fruit rinds (raw drugs) and its value-added products are increasing^4,7^.

*Garcinia* fruits are a rich source of (−)-hydroxycitric acid, anthocyanins and polyisoprenylated benzophenone derivatives like garcinol, camboginol, guttiferones and xanthochymol^10^. (−)-Hydroxycitric acid, often abbreviated HCA in commercial products and advertisements, has gained considerable attention as a promising anti-obesity agent, and commercial *Garcinia* extracts claiming a high content of (−)-hydroxycitric acid are popular food supplements worldwide^11^. In human and animal studies, (−)-hydroxycitric acid has been reported to effectively curb appetite, suppress food intake, increase the rates of hepatic glycogen synthesis, reduce fatty acid synthesis and lipogenesis and decrease body-weight gain^12^. However, contradictory results have been reported, suggesting that (−)-hydroxycitric acid can cause only short-term weight loss, the magnitude of the weight loss effect is small, and the clinical relevance is uncertain^13^. The safety related to the intake of extracts with a high content of (−)-hydroxycitric acid is also uncertain^14^.

A common problem in herbal products highlighted in recent media reports and in scientific literature, is adulteration in high value raw drugs and finished products^15–17^. The underlying reasons for such adulterations may be due to confusion as morphologically similar material identified by vernacular names in different languages enters the chain of commercialization^18^, lack of authentic raw plant material^19^, and also adulteration for financial gain^20^. Adulteration may be considerable, given that there is no regulatory tool for quality control, no strict commercial testing for authentication of herbal products, and a lack of authentication along the value chains for highly traded medicinal plants^21^.

Value chain research focuses on the nature of the relationships among the various participants involved in the chain and on the implications of these relationships for development^21^. The demand for medicinal herbs or herbal products is growing at the rate of 15–25% annually^21^. Surprisingly, only a limited number of studies exist with the focus on value chains for herbal medicines^22^. Understanding and establishing the value chains and trade network for herbal medicines may reflect the reasons for the variation in quality of a particular herbal drug.

To authenticate herbal medicines, different tools for identification are available depending on the plant species and processes involved. These range from straightforward morphological or microscopic identification of plant parts to more advanced genetic or chemical approaches^23^. Among molecular identification tools, DNA barcoding has been successfully used in detecting and quantifying adulteration in raw herbal trade of a variety of medicinal plants. However, the application of the technique is often constrained by the inability to extract good quality DNA from raw herbal materials ranging from simple dried leaves to powdered plant parts, for subsequent PCR-based amplification of DNA barcode markers^19^. Alternatively, spectroscopic methods such as NMR can be used for the analysis of chemical compounds in complex mixtures such as plant extracts, pharmaceuticals and herbal preparations^24^. NMR spectroscopy is highly reproducible, robust and inherently quantitative without the need for prior chromatographic separation of multiple components. The technique has been used for detection, identification and quantitative determination of adulterants in weight loss food supplements^24,25^. Combining DNA barcoding with spectroscopic methods for authentication of herbal medicines increases the resolution in species identification and analysis of mixtures^19^.

In this study, the specific objectives were: (1) to understand the value chain of *Garcinia* species based herbal products; (2) to assess the extent of adulteration in *Garcinia* species in the herbal raw drug trade of Southern India using DNA barcoding; (3) to quantify the principle organic acids, (−)-hydroxycitric acid and (−)-hydroxycitric acid lactone, in *Garcinia* raw herbal drugs using NMR spectroscopy; and (4) to identify and quantify (−)-hydroxycitric acid and (−)-hydroxycitric acid lactone in food supplements that claim to contain *Garcinia* extract, using NMR spectroscopy.

## Materials and Methods

### Raw drug trade analysis

Informal semi-quantitative interviews were conducted in villages of Sirsi (Fig. 1), which is one among the five forest divisions of Uttara Kannada district in Western Ghats of India. Total forest area of Sirsi is 1737 km^2^, and the natural vegetation of Sirsi can be broadly divided into four different categories of forests, viz. tropical evergreen, semi-evergreen, moist deciduous and dry deciduous^26^. Twenty farmers were interviewed about the number of *Garcinia* species available in their region, how many species they collected for trade, and the amounts and which plant parts they collected. They were also asked if they used any post-harvest techniques to process the fruits and if they graded the fruits based on different qualities.

### Collection of authenticated Biological Reference Material

Forty-one taxonomically validated herbarium vouchers from eleven species of *Garcinia* L. were collected from the Western Ghats and Northeast India, (Supplementary Table S1). The collected samples were identified using morphological keys in The Flora of India^27^ as well as by Dr. Srikanth Gunaga, taxonomist at College of Forestry, University of Agricultural Sciences (Sirsi). Nomenclature follows The Plant List (The Plant List, 2013 http://www.theplantlist.org). For each of the species, herbarium specimens were prepared and deposited at the Herbarium of the Ashoka Trust for Research in Ecology and the Environment (ATREE), Bangalore (Supplementary Table S1). These taxonomically authenticated samples are referred to as Biological Reference Material (BRM). The BRM was used to amplify and sequence DNA barcodes for three regions: the nuclear ribosomal nrITS and the chloroplast markers *psbA-trnH* and *rbcL*.

### Raw drug trade samples

**Figure 2:**
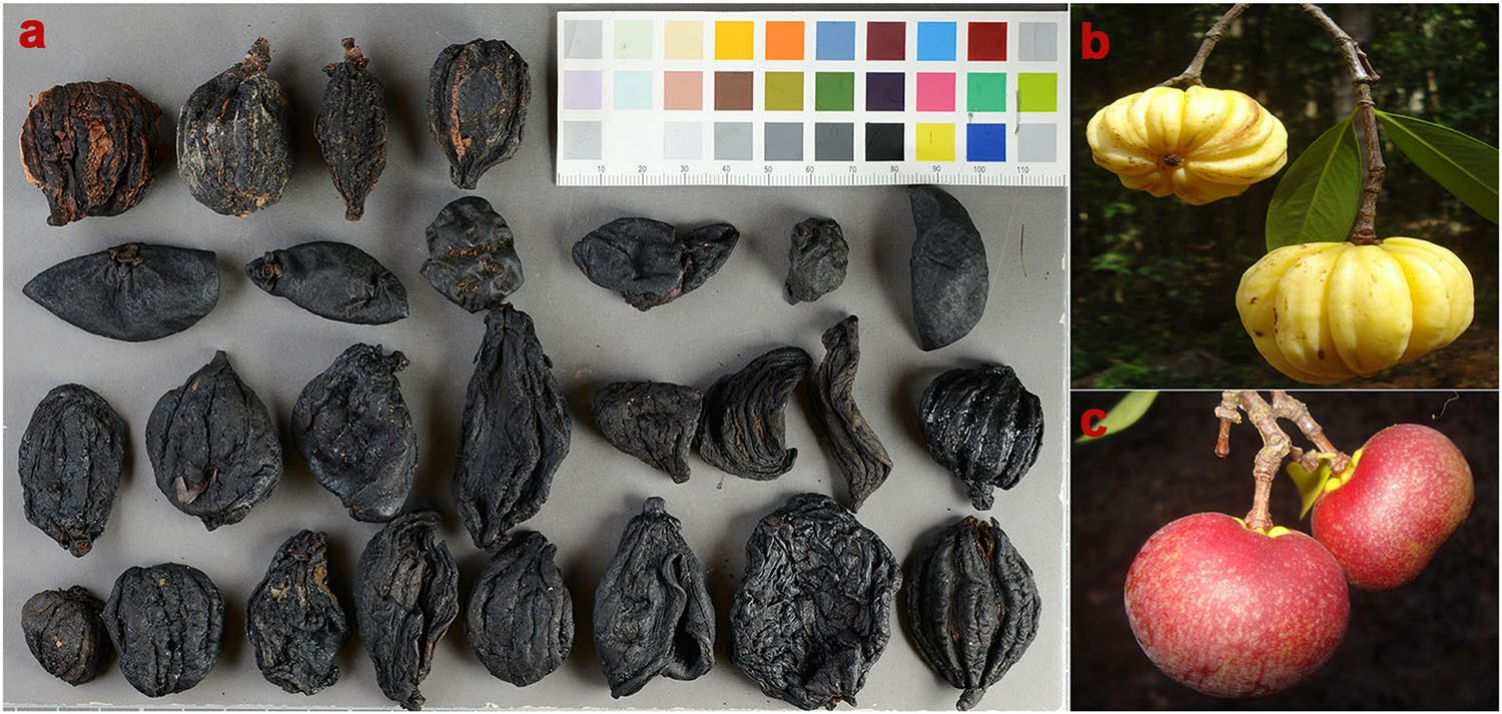
(**a**) Raw drug samples collected as *Kodampuli* and *Kokum* from the raw drug markets of southern India. (**b**) *Garcinia gummi-gutta* fruit. (**c**) *Garcinia indica* fruit.

Raw drug samples were obtained from major raw drug markets in Southern India (Figs 1 and 2). During interviews with vendors we elicited data for all known vernacular and trade names of *Garcinia* species (Supplementary Table S2), but we found that only *Kodampuli* (*G*. *gummi-gutta*) and *Kokum* (*G*. *indica*) were traded. One hundred grams of dried fruit rinds were purchased from each shop, totally 21 shops, and the obtained samples were vouchered as Herbal Authentication Service (HAS) with all the necessary details of shops and place of collection and deposited in the herbarium of ATREE, Bangalore (Supplementary Table S3). A chain of custody protocol ensured that samples were not mixed from the time of collection to DNA extraction and NMR spectroscopic analysis. Although most of the raw drugs collected were dried fruit rinds, they had retained significant morphological features of the fruits.

### Purchase of *Garcinia* herbal products

Ten herbal products that included either *G*. *gummi-gutta* or *G*. *indica* were purchased in pharmacies in India (5), Romania (1), and via e-commerce from United States (2), Norway (1), and Sweden (1). According to the label information, five products contained *G*. *gummi-gutta* (syn. *G*. *cambogia*), two products contained *G*. *indica*, and three products contained *G*. *gummi-gutta*, among other ingredients. An overview of the samples including label information, but not the producer/importer name, lot number, expiration date or any other information that could lead to the identification of that specific product is presented in Supplementary Table S4.

### DNA barcoding and validation of trade samples

DNA was extracted from the leaf tissues of BRM (n = 11 species with 2–5 individuals in each species) using a previously described protocol^28^. Extracted DNA was quantified, and polymerase chain reactions were performed to amplify nrITS, *psbA-trnH* and *rbcL* using specific primers following standard protocols^19,29^ (Supplementary Table S5). The amplified products of nrITS, *psbA-trnH* and *rbcL* were sequenced on an automated ABI 3100 Genetic Analyzer (Applied Biosystems, CA, United States) by Shrimpex Biotech, Chennai, India.

The obtained DNA sequence chromatograms were visualized and edited to remove ambiguous base calls and primer sequences using BioEdit v.5.0.6^30^. The sequences of nrITS, *psbA-trnH* and *rbcL* were deposited in NCBI GenBank and the accession numbers for all the BRM samples are given in Supplementary Table S1. Each species sequence was used as a query sequence in a BLASTn search. Along with the best match sequences in BLASTn search, the available DNA sequences for nrITS and *rbcL* of Indian *Garcinia* species were downloaded in FASTA format from GenBank and included in the analysis. No *psbA-trnH* sequences for these Indian *Garcinia* species were found (Supplementary Table S1). Interspecific and intraspecific genetic distances were calculated in PAUP* v.4.0b.10^31^ using K2P model, as well as the number of parsimony characters per marker. A Maximum Likelihood tree was generated using RAxML^32^ available through the Cipres Web Portal (http://www.phylo.org)^33^. RAxML analyses were run with default parameters for the three markers separately, and *Clusia* species (Clusiaceae) were chosen as outgroup. Further, in order to test the possibilities of concatenating the three markers, incongruence between the nuclear and plastid markers were tested using incongruence length difference test (ILD) in PAUP* v.4.0b.10^31^, and also trees were visually evaluated whether there are any conflicting topologies in the gene trees, defined as ML bootstraps >85 on nodes.

**Figure 3:**
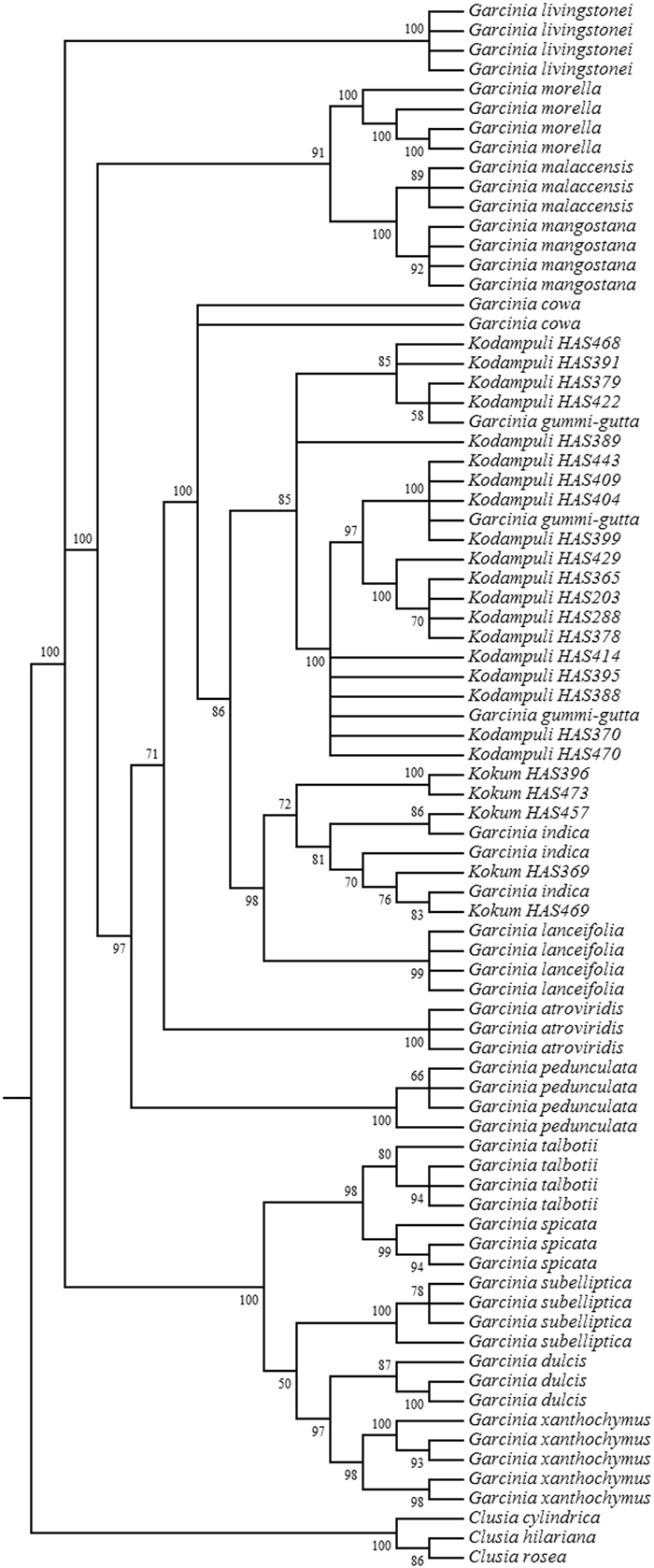
Maximum Likelihood tree (RAxML) of biological reference material of *Garcinia* species and *Garcinia* raw drug market samples using nrITS region.

In order to authenticate identities of the herbal raw drug samples procured as *Kodampuli* and *Kokum*, genomic DNA was isolated using a DNA extraction protocol optimized for *Garcinia* species^28^ and amplified for nrITS and sequenced as described above. A single-locus Maximum Likelihood tree was constructed using both the herbal raw drug samples nrITS sequences and the nrITS BRM barcode library using RAxML^32^ available through the Cipres Web Portal (http://www.phylo.org)^33^ (Fig. 3).

### Preparation of *Garcinia* fruit samples and food supplements for NMR analysis

Due to the highly hygroscopic nature of *Garcinia* fruits, the market samples of *Kodampuli* (n = 18) and *Kokum* (n = 6) were freeze-dried using an Alpha 1–4 LD plus laboratory freeze dryer (Martin Christ GmbH, Osterode am Harz, Germany) for 24 hours and then pulverized in a commercial blender (Waring Commercial, New Hartford, CT, United States). Aliquots of ca. 1 g, accurately weighed, of fruit powder were extracted with Milli-Q water at 120 °C using an Accelerated Solvent Extraction system (ASE350 Solvent Extractor, Dionex, Sunnyvale, CA, United States). Three grams of diatomaceous earth (Dionex) was mixed with the *Garcinia* fruit powder aliquots and loaded in 100 ml steel cartridges. The cartridges were fitted on to the system and exhaustive extraction was performed with three cycles. Preheating time was 5 min, static extraction per cycle was 5 min and the extraction was carried out under a pressure of ca. 1500 PSI (10 MPa). The extracts from each cycle was combined, and water was evaporated at 60 °C using a rotary evaporator (RV 10 Basic Rotavapor with vacuum controller, IKA-Werke GmbH & Co. KG, Staufen, Germany). Further the extract was dried by freeze-drying, yielding 462.2–772.3 mg (45.7–76.6%) of water extract.

For *Garcinia* food supplements analysis, the capsules were opened and their contents removed (7 capsules), whereas tablets were ground to powder (3 tablets). One to two grams of each food supplement was accurately weighed (Supplementary Table S4) and extracted (extraction yield was 0.8758–1.1854 g (53.8–98.3%)). Extraction and NMR analysis were carried out using the same methodology as for the *Garcinia* water extracts.

Apart from water extracts of fruit rinds and food supplements, methanol extracts of *Garcinia* food supplements and *Garcinia* fruit rind powder were prepared following a modified protocol^34^ in order to understand whether polyisoprenylated xanthones can be used as marker compounds for identification. Briefly, aliquots of capsule contents, powdered tablets or fruit rind powder were weighed into 2 ml Eppendorf tubes. CD_3_OD (1.5 ml) was added and the samples were vortexed for 1 min. The samples were then ultrasonicated for 20 minutes at room temperature before centrifugation at 1000 *g* for 30 minutes. To the supernatant was added 0.1% TFA and the acidified samples were transferred to NMR tubes for analysis.

### NMR quantification of (−)-hydroxycitric acid and (−)-hydroxycitric acid lactone

^1^H NMR spectra of *Garcinia* water extracts and food supplement extracts were acquired for quantitative analysis on a Bruker AVII400 (Bruker GmbH, Rheinstetten, Germany) instrument equipped with a 5 mm BBOF probe and operating at a frequency of 400 MHz at 298 K, and with number of scans = 128. A reference solvent solution was prepared containing 2.0 mg/ml maleic acid (internal reference) (Sigma-Aldrich, St. Louis, Missouri, United States) and 0.1% 3-(trimethylsilyl) propionic 2,2,3,3-*d*_4_ acid sodium salt (TSP) (Sigma-Aldrich) in D_2_O (Sigma-Aldrich). For NMR analysis, extracts were dissolved in 500 µl of the NMR reference solvent solution under vortex agitation, giving a final concentration of 20 mg/ml of extract, and analyzed in triplicate. The pH of all *Garcinia* water extracts was measured to have a pH of ∼2 in the NMR solutions. For the *Garcinia* food supplement extracts, the NMR sample solutions were adjusted to a pH of ∼2 by adding trifluoroacetic acid-d (Sigma-Aldrich), which ensures that the carboxylic acid functional groups are in the protonated state and gives consistent shift values for (−)-hydroxycitric acid and (−)-hydroxycitric acid lactone. Shift positions are relative to the TSP signal at δ 0.0 ppm.

The concentrations of (−)-hydroxycitric acid and (−)-hydroxycitric acid lactone were measured by comparing the NMR peak areas of selected protons of (−)-hydroxycitric acid (δ ca. 3.17 ppm, H-3a) and (−)-hydroxycitric acid lactone (δ ca. 3.29 ppm, H-4a) with those of the internal reference (δ 6.37 ppm). The area of each peak is directly proportional to the number of corresponding nuclei. The percentages of (−)-hydroxycitric acid and (−)-hydroxycitric acid lactone in the samples were calculated using the following equation, P (Sample) = (I_(a) × _N_(ir) × _M_(a)_ × m_(ir)_ × P_(ir)_)/(I_(ir)_ × N_(a) × _M_(ir)_ × m_(sample)_), where I(a) and I_(ir)_ are the areas of the analytes ((−)-hydroxycitric acid/(−)-hydroxycitric acid lactone) and the internal reference, N_(ir)_ and N_(a)_ are the number of protons contributing to the signal of the internal reference (2 protons) and the analytes (1 proton each for (−)-hydroxycitric acid and (−)-hydroxycitric acid lactone), M_(a)_ and M_(ir)_ are the molecular weights of (−)-hydroxycitric acid (208.12 g/mol) and (−)-hydroxycitric acid lactone (190.11 g/mol) and the internal reference (116.07 g/mol), m_(ir)_ and m_(sample)_ is the mass of the internal reference (1 mg) and mass of the sample aliquot (10 mg), P_(ir)_ is the purity of the internal reference (99.94%). The areas were measured by using the MNova v. 7.1.2 program (http://mestrelab.com/). In order to evaluate the NMR quantification method, test solutions containing *Garcinia* water extract in NMR reference solvent solution (0.18 to 40 mg/ml) were prepared and measured in triplicates. The S/N ratios were measured to determine Limit of Detection (LOD) and Limit of Quantification (LOQ)^35^, and linearity over the concentration range was also calculated.

### Data accessibility

GenBank (NCBI) [http://www.ncbi.nlm.nih.gov/genbank] Accession numbers of nucleotide sequences are listed in Supplementary Table S1.

## Results and Discussion

### Interviews with *Garcinia* collectors

The interviews with farmers from Western Ghats revealed that people predominantly collected *G*. *gummi-gutta* and *G*. *indica* from the wild, with quantities of *Garcinia* fruits up to 15 to 20 kg per day. Fruits are the traditionally used plant part of *Garcinia*, but during the interview we noticed that resin from the trees was also locally collected^36^. Apart from these two species, *G*. *morella* was collected for culinary purposes and domestic consumption. The other *Garcinia* species such as *G*. *mangostana* and *G*. *cowa*, which grow sparsely in the area, were locally traded based on their seasonal availability. Farmers commented that not all trees produce fruits, and distinguished between “male” and “female” trees of *Garcinia* species. The preliminary understanding from the survey is that there is limited added value for *Garcinia* trade at the farmers end. However, people do process the fruit post-harvest and tend to sell seedless fruits, which fetch a higher price than the fruits with seeds. In the case of *G*. *indica*, the juice is extracted from the fruits by several small and large-scale industries.

### DNA barcoding of *Garcinia* raw drugs

In recent years, a number of medicinal plants have been shown to be adulterated with other low cost plant materials^15^. Contamination in herbal products presents a potential health risk for consumers^23^. In the present study, DNA barcoding was used to analyze the extent of adulteration in herbal raw drug trade of *Garcinia* species. The first step in analyzing the presence of adulteration was the construction of a taxonomically authenticated BRM DNA barcode library for *Garcinia* species. This was assembled in order to provide a reliable DNA library of reference samples, to authenticate the herbal raw drug samples. A single-locus approach was used to delineate nine species collected from Western Ghats and two species from Northeast India based on genetic divergence of three regions (i.e., nrITS, *psbA-trnH* and *rbcL*) using the Kimura 2-Parameter model (Table 1). *rbcL* and *psbA-trnH* exhibited the lowest mean interspecific distance (0.023 and 0.129, respectively); in contrast, nrITS exhibited the highest mean interspecific distance (0.153). Similarly, the mean intraspecific distance for nrITS was 0.037, which was significantly lower than the mean interspecific distance. In the case of *psbA-trnH* and *rbcL*, the mean intraspecific distance was 0.011 and 0.037 respectively, showing that *rbcL* was not a suitable marker because that the interspecific distance, 0.023 is lower than the intraspecific distance, 0.037, and that *psbA-trnH* was considerably better with a significant mean inter- and intraspecific genetic distance (0.129 and 0.037 respectively) (Table 1). nrITS had the most parsimony-informative characters with 278 characters. Similarly *psbA-trnH* and *rbcL* had 198 and 70 respectively (Table 1). The ILD test revealed significant incongruence between the nuclear and plastid markers (P > 0.01), and the visual inspection of phylogenetic trees suggests a topology conflict among the three markers, therefore the three matrices were not concatenated and single-locus trees were utilized for further analyses.

**Table 1:** Evaluation of the three DNA barcode regions used for *Garcinia* species.

DNA regions	Reference PCR success (%)	Aligned sequence (bp)	Intraspecific distance (mean ± SD)	Interspecific distance (mean ± SD)	p-value	Parsimony-informative sites
ITS	100	581	0.037 ± 0.010	0.153 ± 0.085	<0.01	278
*psbA-trnH*	79	493	0.037 ± 0.010	0.129 ± 0.044	<0.01	198
*rbcL*	93	710	0.011 ± 0.005	0.023 ± 0.007	<0.01	70

Authentication of herbal products has been a major challenge due to the fact that chemical studies have documented high content variability of active ingredients of medicinal plants due to geographic conditions and also variability among products from diverse manufacturers of supplements^16^. Conventional techniques such as organoleptic analysis and microscopy are also limited for species authentication in herbal products^37^. There are a number of studies that have assessed adulteration in medicinal plants in raw drug trade and also have identified ingredients added in finished herbal products using DNA barcoding^15,38–40^.

However, a major limitation of Sanger DNA barcoding is the inability to distinguish constituent species in multi-ingredient herbal products^20,41^. Several comprehensive reviews on DNA-based authentication of botanicals have highlighted the merits and limitations of Sanger based DNA barcoding^23,37,42^. In this study, it is evident from the single-locus phylogenetic trees that nrITS and the *psbA-trnH* spacer are more suitable barcode regions for Indian *Garcinia* species than *rbcL* (Supplementary Figs S1, S2, and S3).

Tree-based methods are advantageous over sequence similarity based methods like BLAST that require a decision on a threshold at which a sequence is considered to belong to a certain taxon, which could be subjective and may be applicable to certain taxa but not to others^43^. In tree-based methods, a clear advantage is that no cut-off value is necessary if the query sequence is considered to belong to a certain taxon, and if it is found in the same clade consisting of a reference sequences for that particular taxon^38^. In addition distance based methods (e.g., BLAST) have a tendency to produce false positive identifications if the reference sequences are not available in databases, whereas taxonomic identification based on tree-based methods are not as sensitive to incomplete databases and avoid false positive identification^43^. Supplementary Figs S1 and S2 of nrITS and *psbA-trnH* illustrate the clear grouping within *Garcinia* species with well supported bootstrap values, whereas *rbcL* on the other hand shows poor resolution in delineating the *Garcinia* species, and that can be explained by the low genetic divergence within this marker (Supplementary Fig. S3).

In order to authenticate the raw drugs traded as *Kodampuli* and *Kokum*, the marker nrITS was utilized due to its high amplification success rate from raw drugs (100%), whereas we were unable to amplify the chloroplast markers from the DNA isolated from the raw drug samples. Similarly, it was observed that the PCR amplification success rate in reference samples was 100% for nrITS compared to *psbA-trnH* (79%) and *rbcL* (93%) (Table 1). Analyses of nrITS sequences of *Kodampuli* and *Kokum* along with BRM barcode library revealed that there was no adulteration in the species (Fig. 3), and also that the morphological characters of fruits aided in the authenticity verification of collected raw drug samples from the market (Fig. 2). This was also apparently due to the absence of taxonomic complexities in fruit morphological characters of *Kodampuli* and *Kokum*^44^.

A prerequisite for DNA-based molecular analysis of herbal products is the isolation of good quality DNA^23^. In this study, the yield of isolated DNA from the *Garcinia* food supplements ranged from 0.05 to 1 ng/ml. Plant DNA can also be removed or degraded during the manufacturing process of herbal products: extensive heat treatment, irradiation, ultraviolet light exposure, filtration, extractive distillation or supercritical fluid extraction all affect DNA yield and integrity^23^.

### NMR quantification method in *Garcinia* fruits

**Figure 4:**
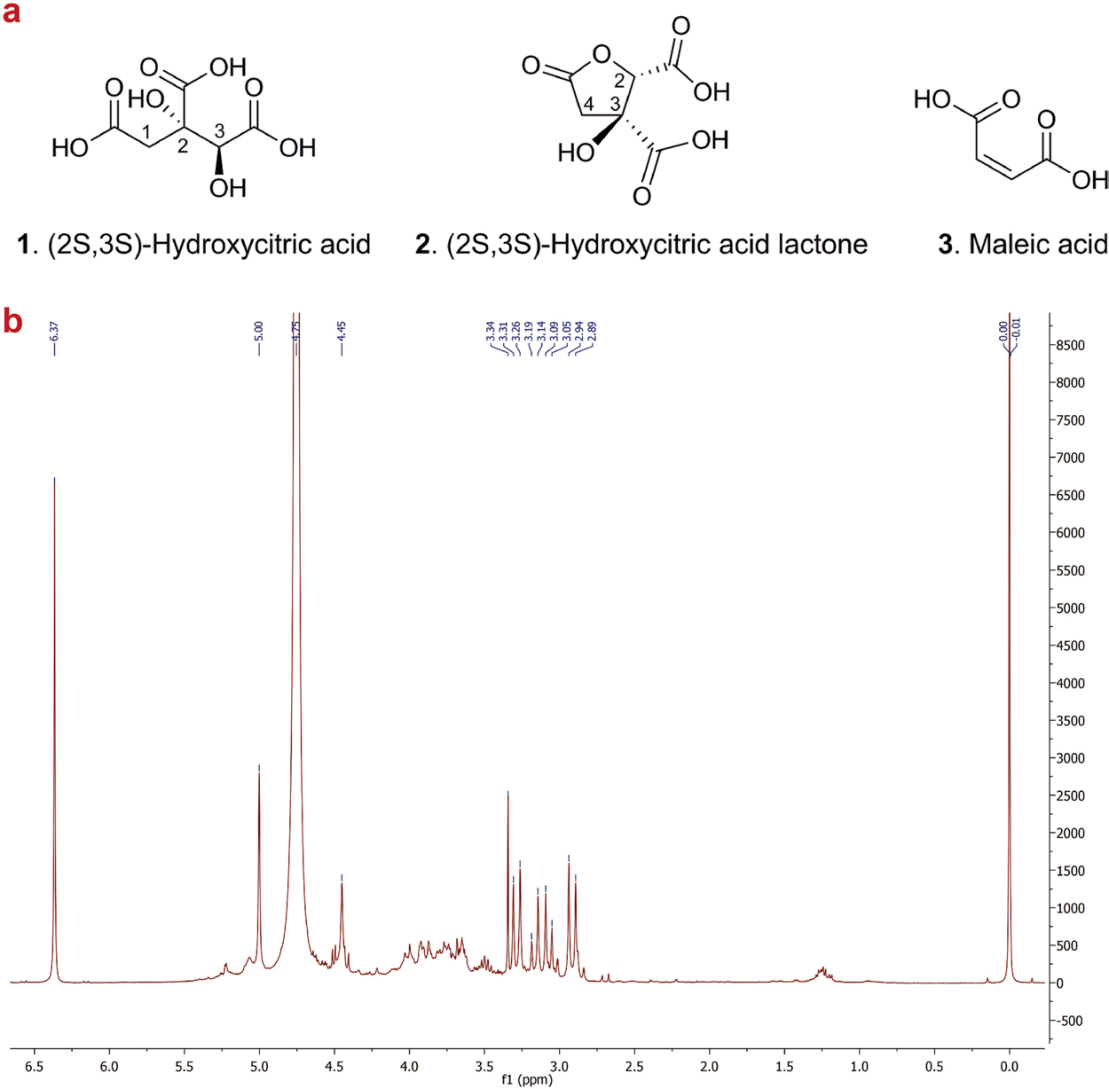
(**a**) Chemical structures of (−)-hydroxycitric acid (**1**), (−)-hydroxycitric acid lactone (**2**) and maleic acid (**3**). (**b**) ^1^H NMR spectrum of *Garcinia gummi-gutta* (voucher no. HAS429) extract.

^1^H NMR was used to quantify the (−)-hydroxycitric acid and (−)-hydroxycitric acid lactone content in raw herbal drugs of *Garcinia* species. The chemical formulas for (−)-hydroxycitric acid with the (2 S,3 S) configuration and its lactone form are shown in Fig. 4a. The (−)-hydroxycitric acid content was directly measured from areas of characteristic signals in the proton spectra of the water extracts (Fig. 4b). Limit of detection (LOD) and limit of quantification (LOQ) is the lowest concentration of an analyte in a sample which can be detected and quantified, respectively, for which the selected signals exhibit the minimal required S/N ratio. Table 2 shows LOD values of 0.06 and 0.04 mg/ml, and LOQ values of 0.13 and 0.20 mg/ml for (−)-hydroxycitric acid and (−)-hydroxycitric acid lactone respectively. Supplementary Fig. S4 shows NMR spectra of *Garcinia* fruit extracts in different concentrations with the resulting changes in signal intensities for (−)-hydroxycitric acid and (−)-hydroxycitric acid lactone.

**Table 2:** Method validation data for quantification of (−)-hydroxycitric acid and (−)-hydroxycitric acid lactone.

Compound	LOD (mg/ml)	LOQ (mg/ml)	Relative standard deviation	Linear equation
(−)-Hydroxycitric acid in *Garcinia* fruit extracts	0.06	0.45	1.3%	y = 1.0143 × +100.92 R² = 0.9374
(−)-Hydroxycitric acid lactone in *Garcinia* fruit extracts	0.04	0.71	2.1%	y = 2.8848 × +163.45 R² = 0.9974

The ^1^H NMR spectrum of the water extract of *G*. *gummi-gutta* HAS429 depicted in Fig. 4b clearly shows the presence of characteristic signals from (−)-hydroxycitric acid (**1**). The ^1^H NMR signals for H-1 appear as two closely spaced doublets at δ 3.07 ppm (d, *J* = 16.6 Hz) and 3.16 ppm (d, *J* = 16.6 Hz), while H-3 appears as a singlet at δ 4.45 ppm. In the same spectrum the H-4 protons in (−)-hydroxycitric acid lactone (**2**) give rise to two doublets at δ 2.92 ppm (d, *J* = 18.0 Hz) and 3.29 ppm (d, *J* = 18.0 Hz), H-2 gives a singlet at δ 5.00 ppm. The two vicinal protons of the internal standard maleic acid (**3**) give rise to the singlet at δ 6.37 ppm. However, a slight variation in the characteristic signals of (−)-hydroxycitric acid and (−)-hydroxycitric acid lactone within triplicates of each sample and between samples was observed (relative standard deviation < 0.03). These variations in chemical shifts values are possibly due to minor pH differences or interactions with other molecules in the extracts.

Though no prior sample clean-up after extraction was necessary in order to quantify the (−)-hydroxycitric acid and (−)-hydroxycitric acid lactone content in the *Garcinia* fruit extracts, it was observed that the extracts are rich in sugar compounds (Fig. 4b & Supplementary Fig. S5). Table 3 shows the percentage of (−)-hydroxycitric acid and (−)-hydroxycitric acid lactone (mass fraction) in the dried fruits of *Kodampuli* and *Kokum* raw drugs. The content of (−)-hydroxycitric acid in the dried *Kodampuli* fruits varied from 3.9% to 10.3% with an average of 7.9% and the content of (−)-hydroxycitric acid lactone varied from 7.4% to 19.1% with an average of 13.1%. On the other hand, the content of (−)-hydroxycitric acid in the dried *Kokum* fruits varied from 1.7% to 7.6% with an average of 4.3% and the content of (−)-hydroxycitric acid lactone varied from 3.5% to 11.7% with an average of 7.8%. Since the equilibrium state of (−)-hydroxycitric acid and (−)-hydroxycitric acid lactone in the *Garcinia* fruits is not known, the total content of the acid and the lactone was calculated. The total average content of (−)-hydroxycitric acid and (−)-hydroxycitric acid lactone in *Kodampuli* was 21.0% while the total content in *Kokum* was 12.1%. As far as we know, this is the first time that ^1^H NMR has been used for the quantification of (−)-hydroxycitric acid and (−)-hydroxycitric acid lactone. Gas chromatography^45^, high performance liquid chromatography^46^, and capillary zone electrophoresis^47^ have previously been used to quantify the hydroxycitric acid and hydroxycitric acid lactone content in *Garcinia* fruits and food supplements. HPLC with UV detection at 210–220 nm is commonly used today and has replaced the more laborious GC methods^12,48^. A challenge with HPLC is to obtain a good resolution between the organic acids and their lactones in *Garcinia*. There are examples showing no discrimination between (−)hydroxycitric acid and the lactone or methods that suffer from poor resolution^46,49,50^. Another challenge is the low selectivity at 210 nm, therefore sample preparation may be needed to remove co−eluting interfering substances^46,49,50^. Compared to these methods, quantitative NMR (qNMR) currently employed has the advantage of being rapid (less than 8 minutes needed for 128 scans) with no need for additional cleanup of extracts or derivatization. NMR makes it possible to detect of all kind of organic molecules in the same sample, as long as they are soluble in the NMR solvent, and is also highly reproducible with little instrument-instrument variation; in addition it is non-invasive and non-destructive^51^.

**Table 3:** (−)-Hydroxycitric acid, and (−)-hydroxycitric acid lactone content (%) of *Garcinia gummi-gutta* (*Kodampuli*) and *Garcinia indica* (*Kokum*) fruit rinds using ^1^H NMR for quantification (*Garcinia indica samples).

Voucher of dried fruits	(−)-Hydroxycitric acid content, % (SD)	(−)-Hydroxycitric acid lactone content, % (SD)	Total (−)-hydroxycitric acid and (−)-hydroxycitric acid lactone content, % (SD)
HAS370	8.5 (2.9)	14.8 (1.4)	23.3 (3.7)
HAS404	8.2 (0.4)	14.4 (0.4)	22.6 (0.8)
HAS391	10.3 (0.6)	19.1 (1.2)	29.4 (1.6)
HAS468	7.6 (0.4)	11.7 (1.0)	19.4 (0.9)
HAS378	6.3 (0.3)	7.8 (0.3)	14.1 (0.4)
HAS288	7.5 (0.2)	11.9 (0.9)	19.5 (1.1)
HAS470	8.8 (0.6)	14.9 (1.2)	23.6 (1.7)
HAS395	7.9 (0.1)	12.5 (0.2)	20.3 (0.1)
HAS388	8.1 (1.1)	14.1 (1.9)	22.2 (3.0)
HAS379	6.7 (0.2)	10.0 (0.5)	16.8 (0.4)
HAS443	8.1 (0.6)	11.3 (0.9)	19.4 (1.4)
HAS422	8.9 (0.4)	17.0 (0.7)	25.9 (1.0)
HAS414	7.8 (0.4)	12.5 (0.6)	20.3 (1.0)
HAS389	8.2 (0.1)	14.4 (1.5)	22.6 (2.5)
HAS409	7.6 (0.4)	13.1 (0.4)	20.7 (0.6)
HAS399	8.9 (0.1)	14.1 (0.2)	23.0 (0.2)
HAS429	8.9 (0.3)	14.5 (0.2)	23.4 (0.5)
HAS203	3.9 (1.3)	7.4 (0.3)	11.3 (1.3)
HAS469*	7.6 (0.3)	11.7 (0.4)	19.3 (0.6)
HAS457*	3.7 (1.3)	11.3 (1.0)	15.0 (2.3)
HAS473*	5.0 (0.8)	7.3 (0.7)	12.3 (1.3)
HAS365*	2.7 (0.4)	5.4 (0.2)	8.2 (0.5)
HAS369*	4.8 (0.1)	8.1 (0.1)	12.8 (0.1)
HAS396*	1.7 (0.2)	3.5 (0.4)	5.2 (0.6)

In previous studies, using an HPLC method, a (−)-hydroxycitric acid content of *G*. *gummi-gutta* of 16–18% was reported, and the leaves and rinds of *G*. *indica* were found to contain 4.1–4.6% and 10.3–12.7%, respectively, whereas only trace amounts of (−)-hydroxycitric acid lactone was reported^46,50^. For *G*. *cowa*, the (−)-hydroxycitric acid content was found to be 1.7, 2.3 and 12.7%, respectively in the fresh leaves, fruits and dried rinds^48^. These overlaps between the total content of (−)-hydroxycitric acid and (−)-hydroxycitric acid lactone in *Garcinia* species suggest that these organic acids should not be used as marker compound to distinguish between *Garcinia* species.

Seasonal and geographic variations in chemical composition have been reported for a number of medicinal plants^52,53^, and also chemical variability along the value chains, harvesting pressure, harvest time, collection of immature plant parts etc., have been reported to influence the composition and concentration of plant constituents^54–56^. The variation in the concentration of (−)-hydroxycitric acid and (−)-hydroxycitric acid lactone in *Kodampuli* and *Kokum* raw drugs can be explained since the raw drugs were obtained from different geographic locations of South India (Fig. 1), and concentration of organic acids can also vary based on the harvest time, as has been shown in other fruits^57,58^. The raw drugs stocked in the herbal markets are normally obtained from a single source or from collectors of a specific region. Enquiries with the traders indicated that supplies are made usually by wholesale agents who in turn obtain raw drugs from sub-agents and collectors, and it is likely that the collectors get the *Kodampuli* and *Kokum* from across the Western Ghats of India. This could explain the variation in the concentration of (−)-hydroxycitric acid and (−)-hydroxycitric acid lactone in the studied *Garcinia* fruits.

### Validation of *Garcinia* food supplements

*G*. *cambogia* (syn. *G*. *gummi-gutta*) or unspecified *Garcinia* extracts rich in (−)-hydroxycitric acid are examples of food supplements freely available in pharmacies, health food stores, and via the internet without any regulations and quality control. In order to authenticate the validity of *Garcinia* products, ten *Garcinia* food supplements were randomly purchased via e-commerce and pharmacies (Supplementary Table S4). One of the major challenges with the authentication of *Garcinia* food supplements is the lack of processing information. The majority of the supplements were not labeled with the solvent used for extraction, and thus the composition of the extracts was difficult to predict, and it is not obvious if they contain water-soluble consituents such as hydroxycitric acid or lipophilic compounds like garcinol and camboginol. The content of (−)-hydroxycitric acid and (−)-hydroxycitric acid lactone in the *Garcinia* supplements is shown in Table 4, and compared with the labeled contents. Product 1 and 5, labeled to contain 500 mg and 525 of extract with 60% HCA, were found to contain only 36 ± 2.9 mg (5.5%) and 29 ± 1.9 mg (4.6%) (−)-hydroxycitric acid, respectively. The other eight products contained more reasonable amounts of (−)-hydroxycitric acid compared to their labeled ingredients (59–289 mg (12.8–50.5%) per capsule or tablet). In comparison the analyzed *G*. *gummi-gutta* fruit rind water extracts were found to contain 32% of (−)-hydroxycitric acid and its lactone on average, and the average content found for *G*. *indica* water extracts was 19%. A previous study utilizing capillary zone electrophoresis concluded that one sample out of five did not contain *G*. *atroviridis* Griff extract as claimed on the label^47^. On the contrary, a study utilizing HPLC revealed no adulteration among *G*. *gummi-gutta* and *G*. *indica* commercial products^59^ and another study using HPLC revealed that *G*. *gummi-gutta* commercial products contained 51–55% of (−)-hydroxycitric acid with small quantities of tartaric, citric, and malic acids^49^. From quantitative NMR analysis of water extracts obtained from the supplements, it was found that five products contained both (−)-hydroxycitric acid and (−)-hydroxycitric acid lactone. Among these, only one product contained quantifiable amounts of (−)-hydroxycitric acid lactone, whereas (−)-hydroxycitric acid lactone was missing or only present in trace amounts in the remaining products (Table 4 & Supplementary Fig. S6). The absence of (−)-hydroxycitric acid lactone in the supplements with *Garcinia* extracts might be due to the extraction method employed^60^.

**Table 4:** (−)-Hydroxycitric acid, and (−)-hydroxycitric acid lactone content in *Garcinia* food supplements used in the study.

Herbal products code no.	Labeled content per capsule/tablet		(−)-Hydroxycitric acid content (mg/capsule) determined by qNMR
	Garcinia ingredients	mg HCA^$^	
1	*G*. *cambogia* extract (60% HCA), 500 mg	300	36 ± 2.9
2	*G*. *cambogia* extract (50% HCA), 500 mg	250	149 ± 0.7
3	*G*. *indica* extract, 350 mg		122 ± 9.2
4	*G*. *cambogia*, 100 mg		59 ± 1.4
5	*G*. *cambogia* extract (60% HCA), 525 mg	315	29 ± 1.9
6	*G*. *cambogia* extract (65% HCA), 250 mg *G*. *cambodia* powder, fruit rind and leaf (1% HCA), 350 mg	166	144 ± 3.7^#^
7	*G*. *cambogia* extract, 300 mg		69 ± 1.8*
8	*G*. *indica* powder, 400 mg		184 ± 7.3
9	*G*. *cambogia* extract, 350 mg		150 ± 4.3
10	*G*. *cambogia* fruit (60% HCA), 500 mg	300	289 ± 8.6

^$^Calculated based on labeled content of HCA; ^#^Contained 41 ± 0.6 mg (−)-hydroxycitric acid lactone, the other samples contained only detectable amounts or no lactone; *Samples were analyzed only twice.

^1^H NMR spectra of *G*. *indica* and G. *gummi-gutta* (syn. *G*. *cambogia*) fruit rind methanol extracts showed characteristic signals from polyisoprenylated xanthones (garcinol or camboginol). Signals from aromatic protons at δ 6.7, 7.0 and 7.2 ppm and unsaturated tertiary methyls groups at δ 1.5–1.7 ppm were observed^61,62^. These signals were also observed in product no. 6, labeled to contain both *G*. *cambogia* extract (250 mg) and powder of fruit rinds and leaves (350 mg), while methanol extracts of the other supplements did not give rise to signals from garcinol or camboginol. These signals were not present in spectra of the fruit rind or herbal supplement water extracts. Thus, garcinol or camboginol, which are likely not present in the *Garcinia* commercial extracts, should not be used as authentication compounds for *Garcinia* supplements.

## Conclusions

In this study, we used DNA barcoding and ^1^H NMR spectroscopy to authenticate *Garcinia* fruits and food supplements. ^1^H NMR is useful for qualitative and quantitative analysis of constituents in medicinal plants and herbal products^24^. However, NMR has, so far, not received a wider application in the official food supplements testing and medicine control laboratories than other chromatographic methods, mostly because the NMR technique was judged to be complicated and instruments too expensive^25^. However, several improvements in instrumentation during the last decade have made NMR available for routine applications^25^. In the future, a metabolomic approach using NMR would be useful for large-scale analysis of *Garcinia* food supplements and this study has shown that qNMR is a fast and sensitive enough method for the quantification of (−)-hydroxycitric acid content. Several studies have advocated the use of DNA barcoding in herbal product authentication and pharmacovigilance^19,23,29,63^ due to its cost effectiveness and ability to identify plant species. In this study, DNA barcoding was used to demonstrate that there was no adulteration or substitution of *Garcinia* species in major herbal markets of south India. These results show the usefulness of DNA barcoding and ^1^H NMR spectroscopy for use in complementing traditional methods of quality control for consumer safety, and demonstrates that DNA barcoding can be used as a screening method for the identification of *Garcinia* species in herbal trade, and NMR for detection and quantification of hydroxycitric acid and (−)-hydroxycitric acid lactone in *Garcinia* fruits and food supplements.

Our ^1^H NMR results suggest that in order to increase consumer confidence by advocating and promoting a higher standard of quality in herbal products, there is an urgent need to study food supplements derived from traditional medicinal plants.

## Electronic supplementary material

Supplementary Information
