# Supplementary material for: Authentication of Garcinia fruits and food supplements using DNA barcoding and NMR spectroscopy
Source: Sci Rep. 2018 Jul 12;8:10561. doi: 10.1038/s41598-018-28635-z (PMC6043575; doi:10.1038/s41598-018-28635-z)

Authentication of *Garcinia* fruits and food supplements using DNA barcoding and NMR spectroscopy

Gopalakrishnan Saroja Seethapathy, Margey Tadesse, Santhosh Kumar J. Urumarudappa, Srikanth Gunaga, Ramesha Vasudeva, Karl Egil Malterud, Ramanan Uma Shaanker, Hugo J. de Boer, Gudasalamani Ravikanth*, and Helle Wangensteen*

*Gudasalamani Ravikanth (gravikanth@atree.org)

*Helle Wangensteen (helle.wangensteen@farmasi.uio.no)

Supplementary Information

Supplementary Table S1. Details of biological reference material samples collected from different parts of South India used in the analysis.

Supplementary Table S2. List of trade/vernacular names used during the collection of *Garcinia* raw drugs samples from raw drug markets of South India.

Supplementary Table S3. Details of raw drug samples collected from different parts of South India used in the analysis.

Supplementary Table S4. Details of *Garcinia* food supplements used in the study.

Supplementary Table S5. Details of the barcode primers used in the study.

Supplementary Table S6. ^{1}H NMR shifts of (2S, 3S)-Hydroxycitric acid observed in the water extract of *G. gummi-gutta* HAS429 depicted in Figure 4b.

Supplementary Table S7. ^{1}H NMR shifts of (2S, 3S)-Hydroxycitric acid lactone observed in the water extract of *G. gummi-gutta* HAS429 depicted in Figure 4b.

Supplementary Fig. S1. Maximum Likelihood tree (RAxML) of *Garcinia* species using nrITS.

Supplementary Fig. S2. Maximum Likelihood tree (RAxML) of *Garcinia* species using *psbA-trnH.*

Supplementary Fig. S3. Maximum Likelihood tree (RAxML) of *Garcinia* species using *rbcL.*

Supplementary Fig. S4. ^{1}H NMR spectrum of (-)-hydroxycitric acid, and (-)-hydroxycitric acid lactone in different concentration of *Garcinia* fruits.

Supplementary Fig. S5. ^{1}H NMR spectrum of *Garcinia* fruits extract.

Supplementary Fig. S6. ^{1}H NMR spectrum of analyzed *Garcinia* food supplements.

Supplementary Table S1. Details of biological reference material samples collected from different parts of South India used in the analysis.

Sl. no.	Species name	Voucher no.	Collection site	GenBank Accession numbers		
				ITS	psbA-trnH	rbcL
1	*Garcinia gummi-gutta* (L.) Roxb.	ATREE119	Ramagondanahalli (FRLHT), Bangalore	KP318329	KP318368	KP318400
		ATREE120	Vaddi ghat, Uttara kannada	KP318330	KP318369	KP318431
		ATREE121	Sirsi, Karnataka	KP318331	KP318370	KP318432
		ATREE122		KP318332	KP318371	KP318401
		ATREE123		KP318333	-	-
2	*Garcinia xanthochymus* Hook.f. ex T.Anderson	ATREE144	Ramagondanahalli (FRLHT), Bangalore	KP318354	KP318387	KP318428
		ATREE145	Chikka Bommasandra (GKVK, Bangalore)	KP318355	KP318388	KP318429
		ATREE146	Sirsi, Karnataka	KP318356	KP318389	KP318433
		ATREE147	Nilkund, Uttara kannada	KP318357	KP318390	KP318430
		ATREE148	Sirsi, Karnataka	KP318358	-	-
3	*Garcinia indica* (Thouars) Choisy	ATREE124	Salkani, Uttara kannada	KP318334	KP318372	KP318402
		ATREE125	Ramagondanahalli (FRLHT), Bangalore	KP318335	KP318373	KP318403
		ATREE126	Chikka Bommasandra (GKVK, Bangalore)	KP318336	KP318374	KP318404
		ATREE127	Sirsi, Karnataka	KP318337	-	KP318405
		ATREE128		KP318338	-	-
4	*Garcinia morella* (Gaertn.) Desr.	ATREE139	Sirsi, Karnataka	KP318349	KP318385	KP318415
		ATREE140		KP318350	KP318386	KP318416
		ATREE141		KP318351	-	KP318417
		ATREE142		KP318352	-	KP318418
		ATREE143		KP318353	-	KP318419
5	*Garcinia mangostana* L.	ATREE137	Sirsi, Karnataka	KP318347	KP318383	KP318413
		ATREE138		KP318348	KP318384	KP318414
6	*Garcinia talbotii* Raizada ex Santapau	ATREE150	Vaddi ghat, Uttara kannada	KP318360	KP318392	KP318434
		ATREE151	Sirsi, Karnataka	KP318361	KP318393	KP318425
		ATREE152		KP318362	KP318394	KP318426

		ATREE153		KP318363	KP318395	KP318427
7	*Garcinia livingstonei* T.Anderson	ATREE154	Sirsi, Karnataka	KP318364	KP318396	KP318410
		ATREE155		KP318365	KP318397	KP318411
		ATREE156		KP318366	KP318398	KP318435
		ATREE157		KP318367	-	KP318412
8	*Garcinia pedunculata* Roxb. ex Buch.-Ham.	ATREE133	Jorhat, Assam	KP318343	KP318379	KP318420
		ATREE134		KP318344	KP318380	KP318421
		ATREE135		KP318345	KP318381	KP318422
		ATREE136		KP318346	KP318382	KP318423
9	*Garcinia lanceifolia* Roxb.	ATREE129	Jorhat, Assam	KP318339	KP318375	KP318406
		ATREE130		KP318340	KP318376	KP318407
		ATREE131		KP318341	KP318377	KP318408
		ATREE132		KP318342	KP318378	KP318409
10	*Garcinia spicata* Hook.f.	ATREE149	Ramagondanahalli (FRLHT), Bangalore	KP318359 EU128390.1*	KP318391	KP318424
		ATREE159	Sirsi, Karnataka	EU128389.1*	KP318399	HQ332063.1*
11	*Garcinia cowa* Roxb. ex Choisy	ATREE158	Sirsi, Karnataka	AF367213.1* AB110799.1*	-	KJ510948 *

*Sequences downloaded from NCBI

Supplementary Table S2. List of trade/vernacular names used during the collection of *Garcinia* raw drugs samples from raw drug markets of South India.

Sl. no.	Species name	Trade name /Vernacular name
1	*Garcinia gummi-gutta* (L.) Roxb.	Kodampuli/ kodukkappuli
2	*Garcinia xanthochymus* Hook.f. ex T.Anderson	Deavkai/ malaippuli
3	*Garcinia indica* (Thouars) Choisy	Kukam/ kokam
4	*Garcinia morella* (Gaertn.) Desr.	Kadukaai puli/ punarpuli
5	*Garcinia mangostana* L.	Shulampuli/ mangosteen
6	*Garcinia talbotii* Raizada ex Santapau	Tavir
7	*Garcinia livingstonei* T.Anderson	-
8	*Garcinia pedunculata* Roxb. ex Buch.-Ham.	Nerinnampuli
9	*Garcinia lanceifolia* Roxb.	-
10	*Garcinia spicata* Hook.f.	Kaadu jaarige
11	*Garcinia cowa* Roxb. ex Choisy	Dvipaja/ paravata

Supplementary Table S3. Details of raw drug samples collected from different parts of south India used in the analysis.

Sl. No	Location of Shops/State	Kodampuli	Kokum
1.	Salem/Tamil Nadu	HAS443	-
2.	Vellore/Tamil Nadu	HAS429	-
3.	Panruti/Tamil Nadu	HAS414	-
4.	Villupuram/Tamil Nadu	HAS409	-
5.	Cuddalore/Tamil Nadu	HAS404	-
6.	Pondicherry/Tamil Nadu	HAS399	-
7.	Trichy/Tamil Nadu	HAS379	-
8.	Coimbatore/Tamil Nadu	HAS378	-
9.	Tambaram/Tamil Nadu	HAS203	-
10.	Pattukkottai/Tamil Nadu	HAS288	-
11.	Davangere/Karnataka	HAS365	-
12.	Sirsi/Karnataka	HAS370	HAS369
13.	Bangalore/Karnataka	HAS395	HAS396
14.	Virajpet/Karnataka	HAS470	HAS469
15.	Kannur/Kerala	HAS388	-
16.	Thalassery/Kerala	HAS389	-
17.	Thiruvanathapuram/Kerala	HAS391	-
18.	Wayanad/Kerala	HAS422	-
19.	Kaviyoor/Kerala	HAS468	-
20.	Kolhapur/Maharashtra	-	HAS473
21.	Mumbai/Maharashtra	-	HAS457

Supplementary Table S4. Details of *Garcinia* herbal products used in the study.

Herbal products code no.	Species on label	Scientific names of the plant ingredients as indicated in the product	Product type	Country of origin	Country of acquisition	Vendor type	Product classification as indicated in the product	Remarks from label	Weight of capsules/tablets used in the study
1	1	*Garcinia cambogia*	Capsules	USA	India	Pharmacy	Dietary supplement	Each capsule contains *Garcinia cambogia* extracts 500 mg, containing 60% HCA. Potassium 50 mg, calcium 50 mg	1.4481 g (2 capsules)
2	1	*Garcinia cambogia*	Capsules	USA	India	Pharmacy	Dietary supplement	Each capsule contains *Garcinia cambogia* extracts 500 mg, containing 50% HCA. Calcium 160 mg	1.4677 g (2 capsules)
3	1	*Garcinia indica*	Capsules	India	India	Pharmacy	Not stated	Each capsule contains 350 mg of *Garcinia indica* extract	1.3220 g (3 capsules)
4	4	*Commiphora mukul, Garcinia gummi-gutta, Allium sativum Plumbago zeylanica*	Capsules	India	India	Pharmacy	Not stated	*Garcinia gummi-gutta* 100 mg	1.3940 g (3 capsules)
5	1	*Garcinia cambogia*	Capsules	USA	USA	Internet	Dietary supplement	*Garcinia cambogia* extract 1050 mg, standardized to 60% HCA Potassium 160 mg	1.2608 g (2 capsules)
6	1	*Garcinia cambogia* Desr.	Tablets	India	USA	Internet	Herbal Supplement	*Garcinia cambogia* extract 250 mg, standardized to 65% HCA. Garcinia powder 350 mg (fruit rind and leaf), 1% HCA	1.2243 g (2 tablets)
7	5	*Garcinia cambogia Gymnema sylvestre Terminalia chebula Trigonella foecum-graeceum Balsamodendron mukul*	Capsules	Indua	Romania	Pharmacy	Not stated	Extracts*: Garcinia cambogia* (300 mg), *Gymnema sylvestre* (10 mg), *Terminalia sylvestre* (10 mg), *Trigonella foecum-graeceum* (10 mg) Powder: *Balsamodendron mukul* (70 mg)	1.3445 g (3 capsules)
8	2	*Garcinia indica Commiphora mukul,*	Tablets	India	India	Pharmacy	Not stated	Each tablet contains 400 mg powders of *Garcinia indica,*	1.6856 g (3 tablets)

		Emblica officinalis, *Terminalia bellirica*, *Terminalia chebula*						50 mg of Shuddha Guggul (*Commiphora mukul*),<br>90 mg of Triphala Ghan (Three myrobalons)	
9	1	*Garcinia indica*	Tablets	India	Sweden	Internet	Not stated	Each capsule contains 350 mg of *Garcinia indica* extract	1.2419 g (2 tablets)
10	1	*Garcinia cambogia*	Capsules	USA	Norway	Internet	Herbal Supplement	*Garcinia cambogia* fruit 500 mg, standardized to 60% HCA	1.1432 g (2 capsules)

Supplementary Table S5. Details of the barcode primers used in the study.

DNA barcode regions	Primer name	Sequence (5′-3′)	Reference
nrDNA-*ITS*	ITS1	TCCGTAGGTGAACCTGCGG	[1]
	ITS4	TCCTCCGCTTATTGATATGC	
psbA-trnH	psbA	GTTATGCATGAACGTAATGCTC	[2]
	trnH	CGCGCATGGTGGATTCACAAATC	
*rbc*L	F2N	CCAAGTTGAGAGAGATAAATTGAACAAG	[3]
	1460R	TCCTTTTAGTAAAAGATTGGGCCGAG	

References

1. White TJ, Bruns T, Lee S, Taylor J (1990) Amplification and direct sequencing of fungal ribosomal RNA genes for phylogenetics. In M. A. Innis, D. H. Gelfand, J. J. Sninsky, & J. Thomas (Eds.), PCR protocols: A guide to methods and applications (pp. 315). San Diego: Academic Press.
2. Shaw J, Lickey EB, Beck JT, Farmer SB, Liu WS, Miller J, Siripun KC, Winder CT, Schilling EE, Small RL (2005) The tortoise and the hare. II. Relative utility of 21 non coding chloroplast DNA sequences for phylogenetic analysis. Am J Bot 92: 142–166.
3. Fay MF, Bayer C, Alverson WS, de Bruijn AY, Chase MW (1998) Plastid rbcL sequence data indicate a close affinity between Diegodendron and Bixa. Taxon 43-50.

Supplementary Table S6. ^{1}H NMR shifts of (2S, 3S)-Hydroxycitric acid observed in the water extract of *G. gummi-gutta* HAS429 depicted in Figure 4b.

Number	δ_H (multiplicity, *J* in Hz)
1a	3.07 (d, *J* = 16.6 Hz)
1b	3.16 (d, *J* = 16.6 Hz)
3	4.45 (s)

Supplementary Table S7. ^{1}H NMR shifts of (2S, 3S)-Hydroxycitric acid lactone observed in the water extract of *G. gummi-gutta* HAS429 depicted in Figure 4b.

Number	δ_H (multiplicity, *J* in Hz)
2	5.00 (s)
4a	2.92 (d, *J* = 18.0 Hz)
4b	3.29 (d, *J* = 18.0 Hz)

Supplementary Fig. S1. Maximum Likelihood tree (RAxML) of *Garcinia* species using nrITS.

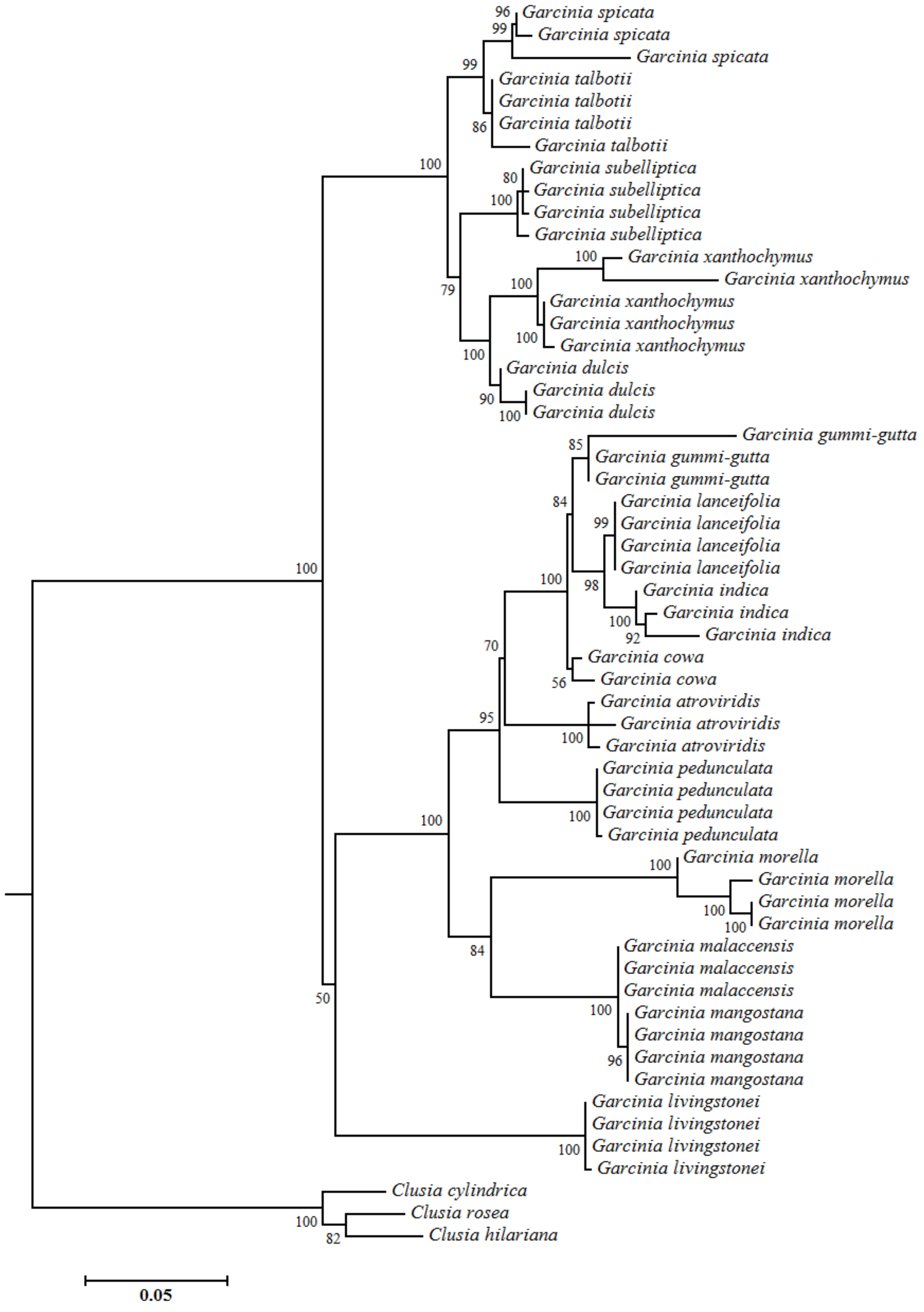

Supplementary Fig. S2. Maximum Likelihood tree (RAxML) of *Garcinia* species using *psbA-trnH*.

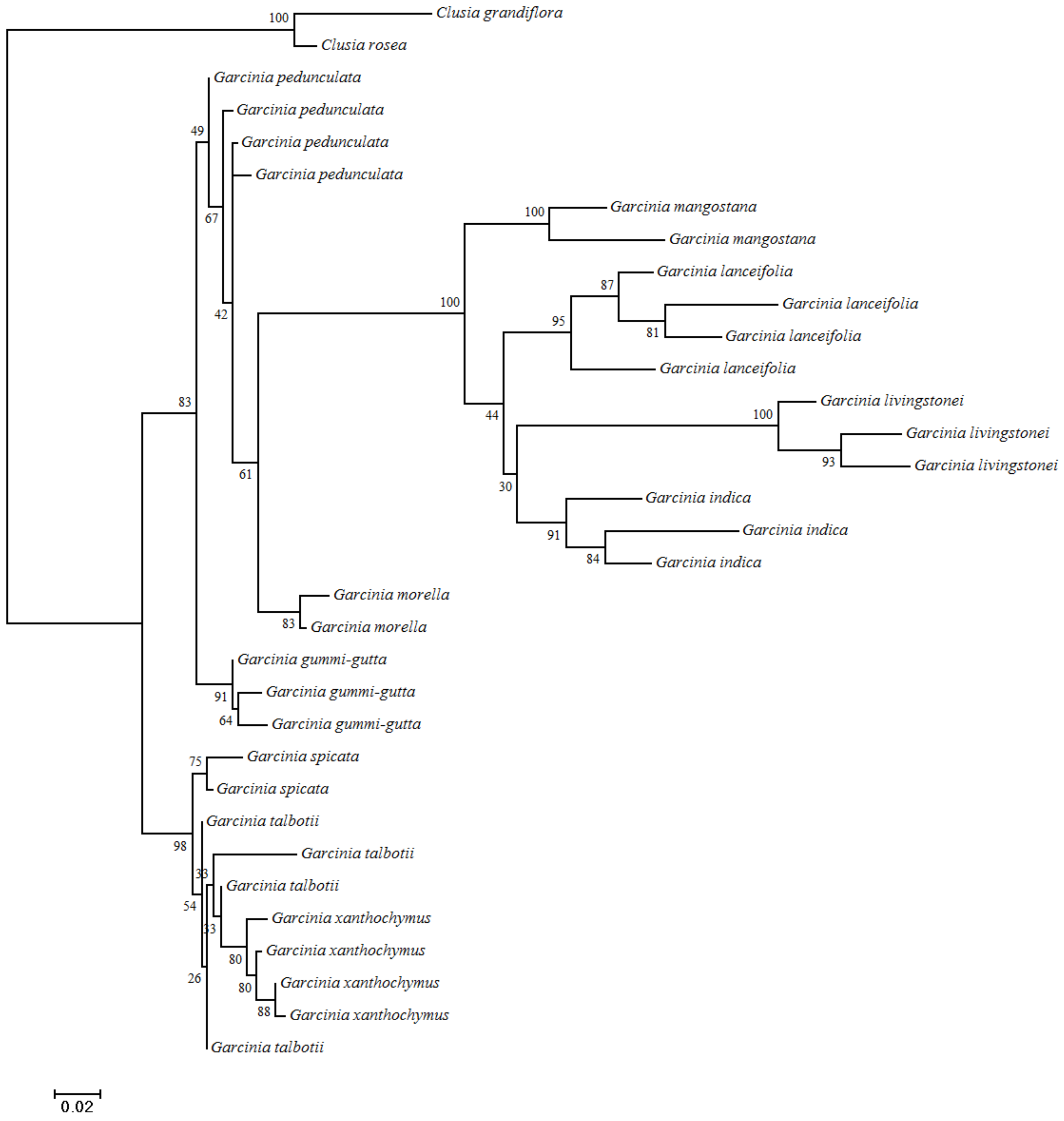

Supplementary Fig. S3. Maximum Likelihood tree (RAxML) of *Garcinia* species using *rbcL*.

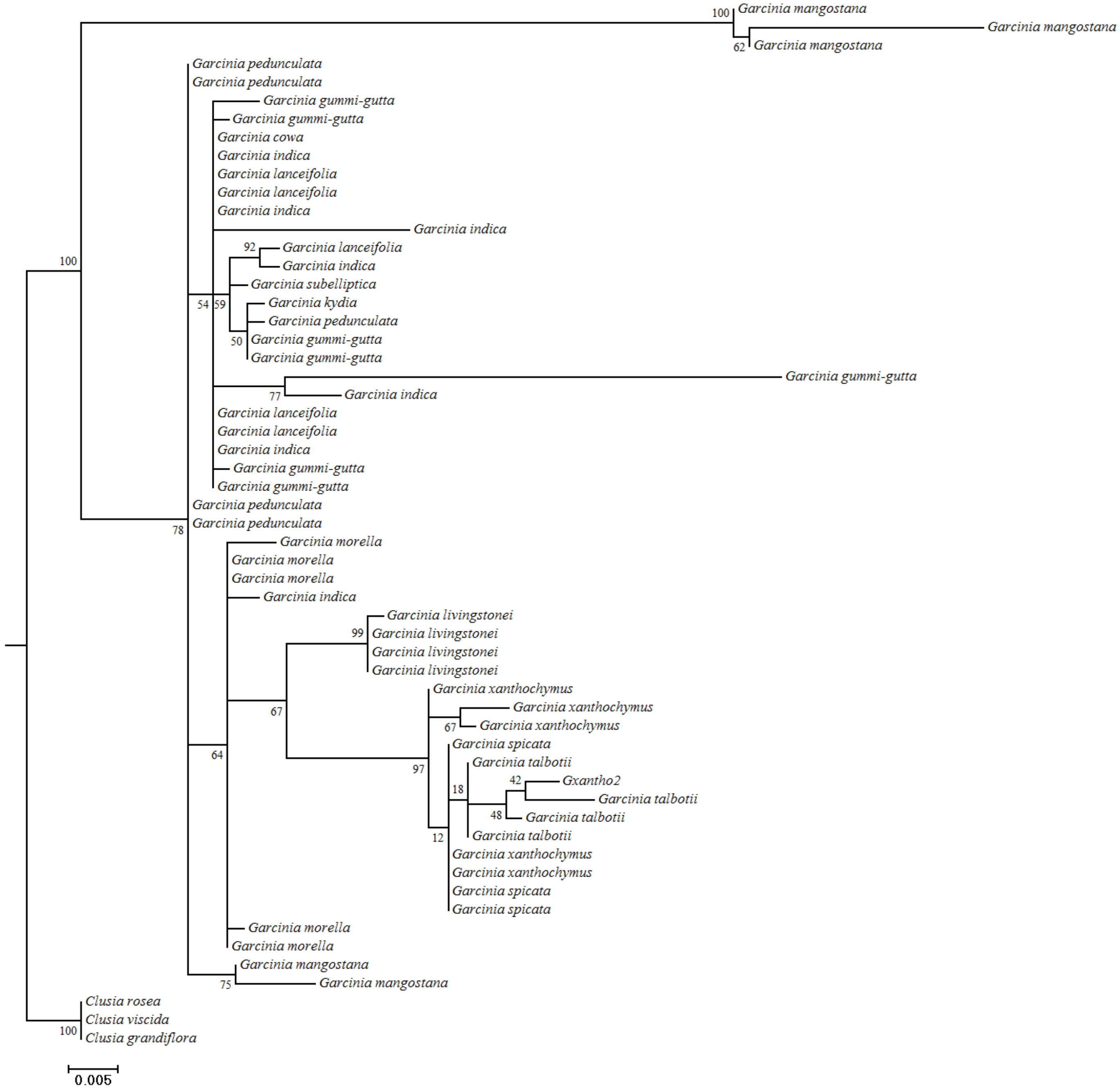

Supplementary Fig. S4. ^{1}H NMR spectrum of (-)-hydroxycitric acid, and (-)-hydroxycitric acid lactone in different concentration of *Garcinia* fruits

Supplementary Fig. S4. Garcinia fruit extract (40 mg/ml)

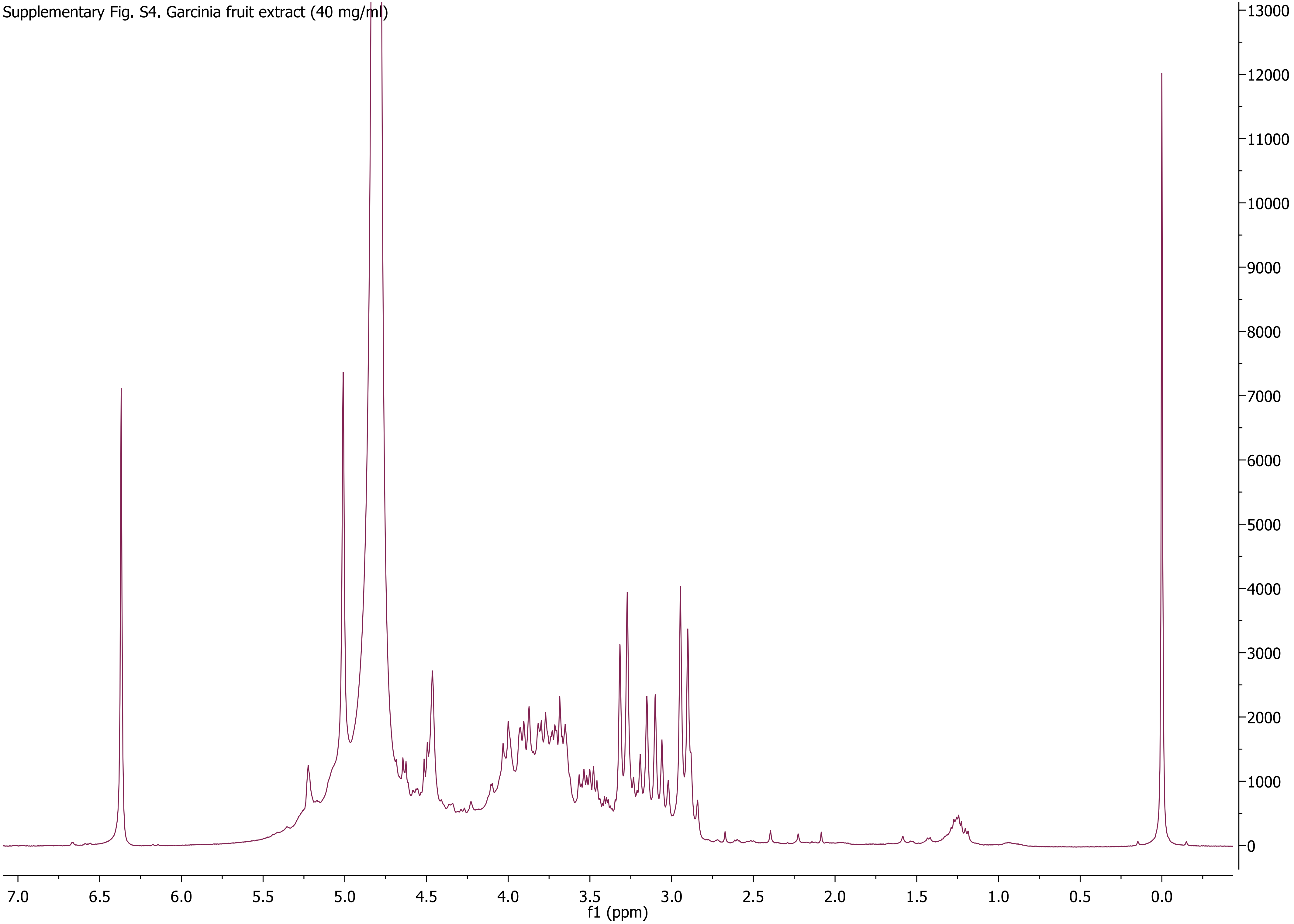

Supplementary Fig. S4. Garcinia fruit extract (20 mg/ml)

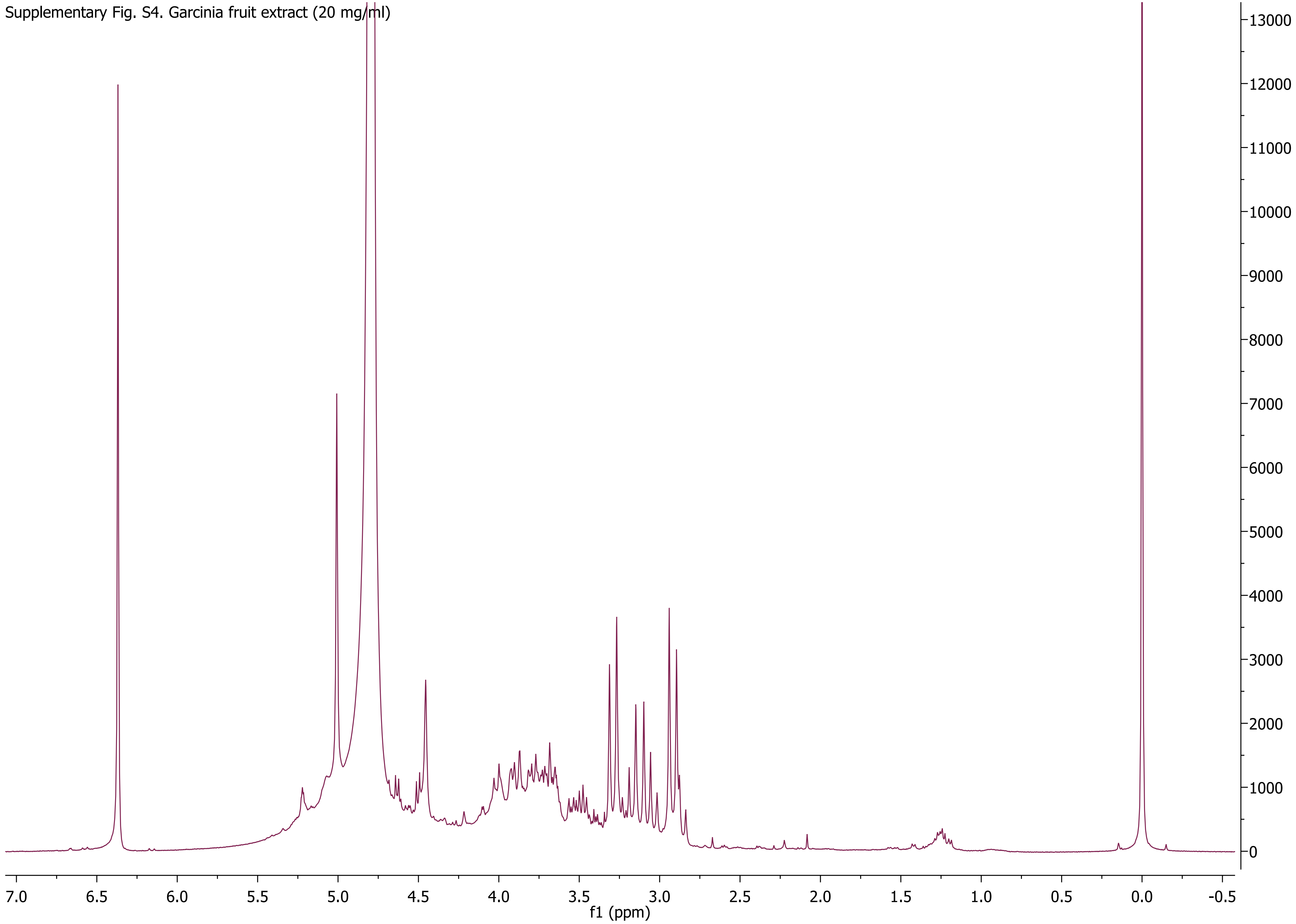

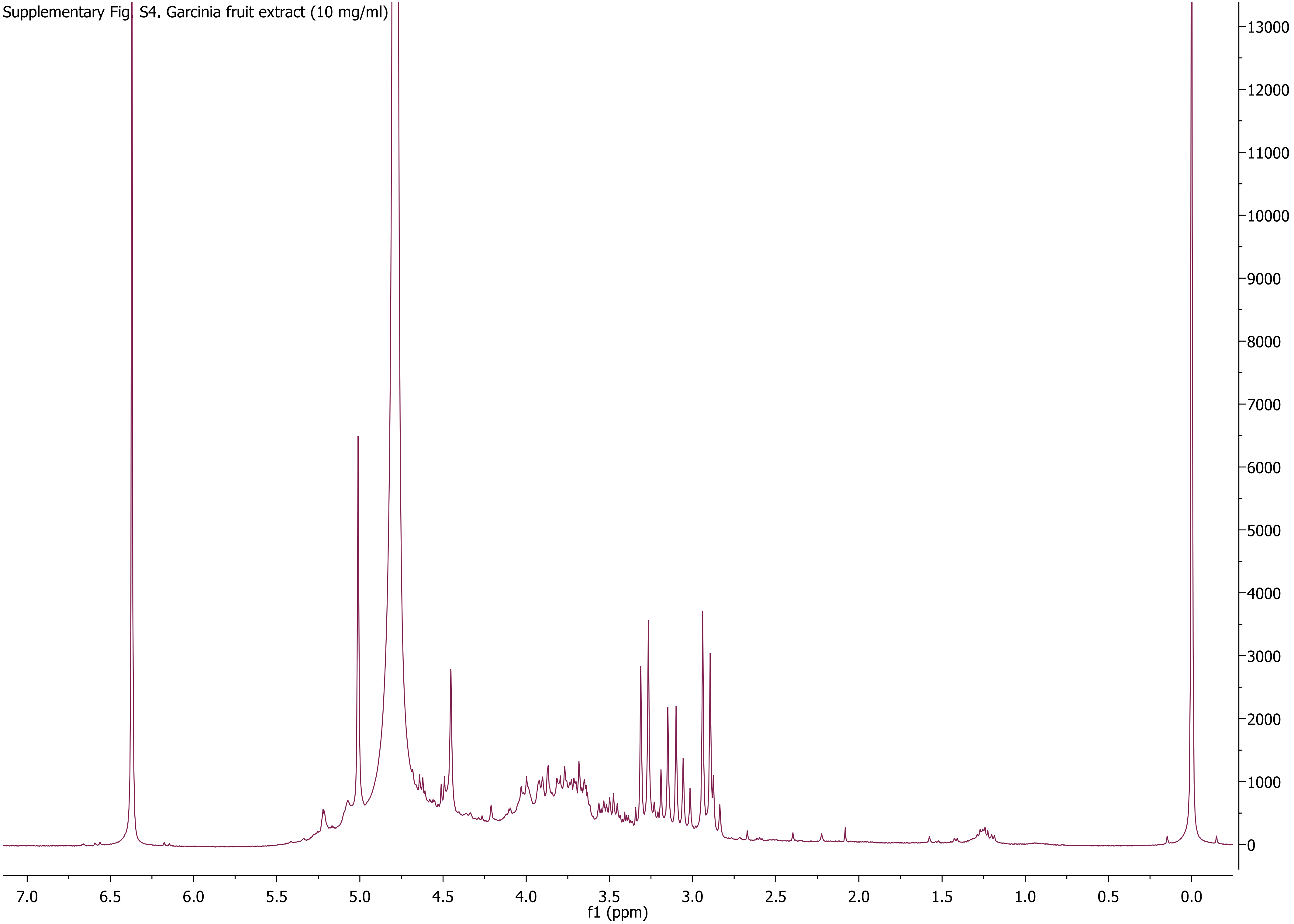

Supplementary Fig. S4. Garcinia fruit extract (10 mg/ml)

Supplementary Fig. S4. Garcinia fruit extract (5 mg/ml)

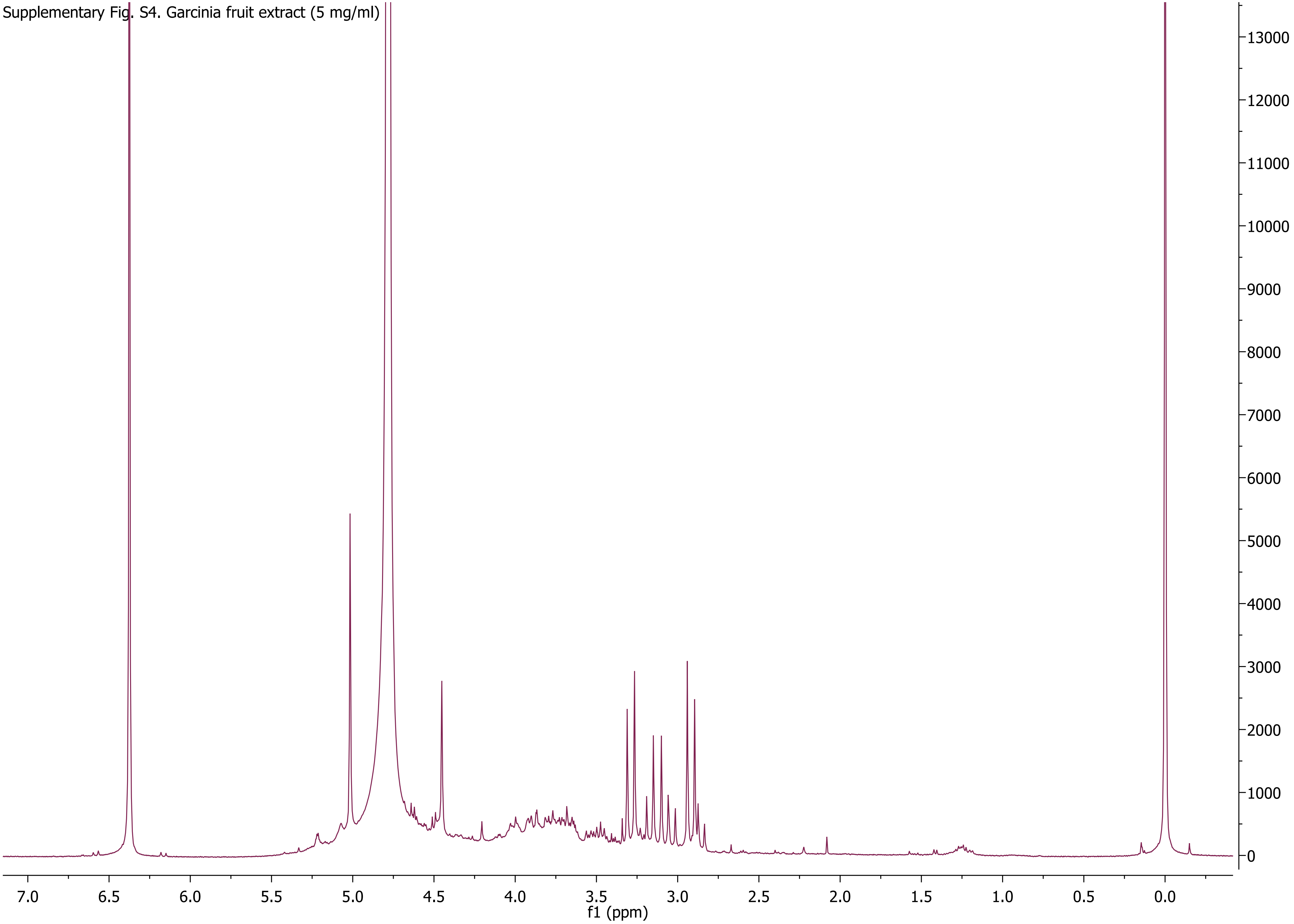

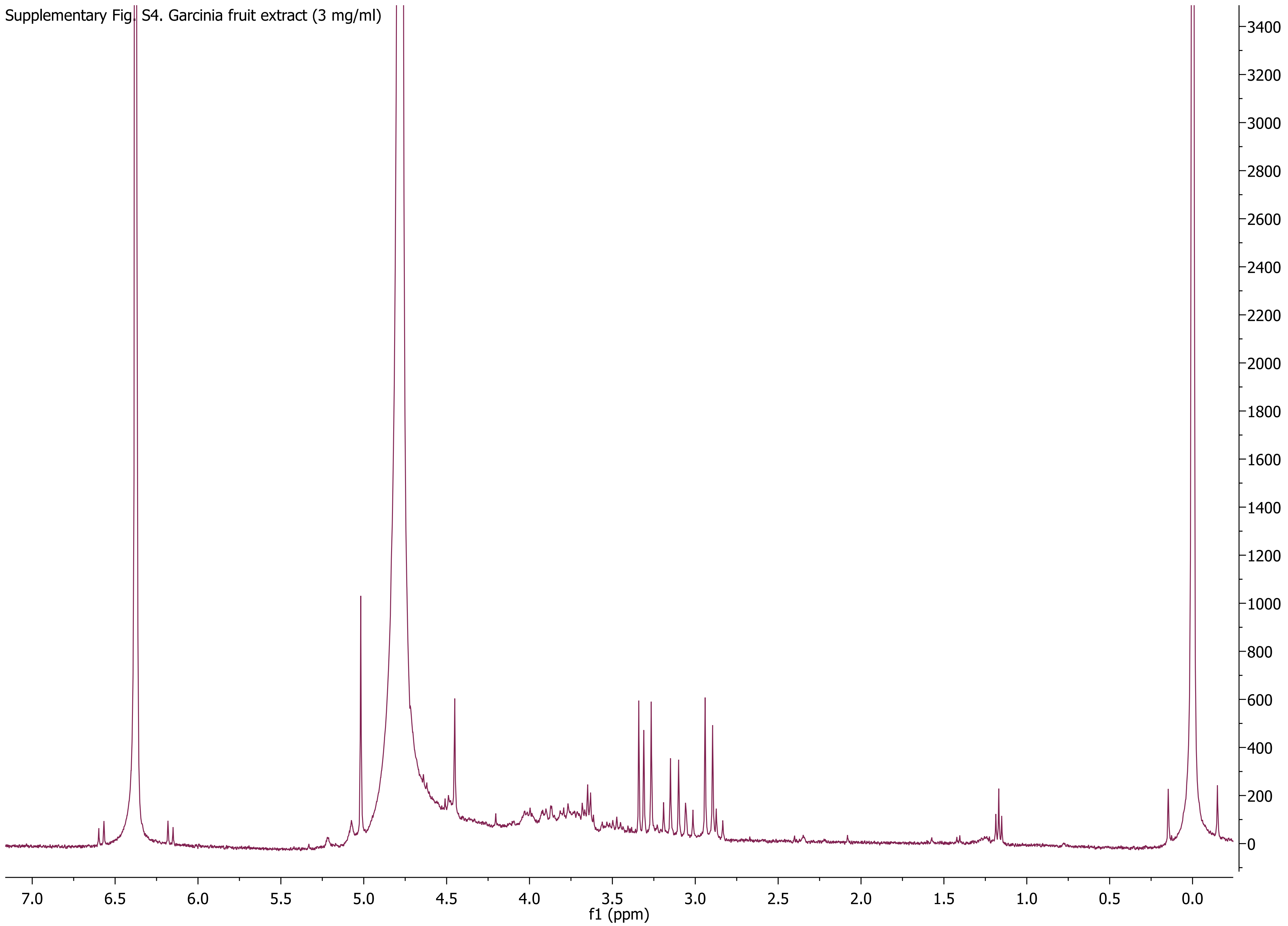

Supplementary Fig. S4. Garcinia fruit extract (3 mg/ml)

Supplementary Fig. S4. Garcinia fruit extract (1.5 mg/ml)

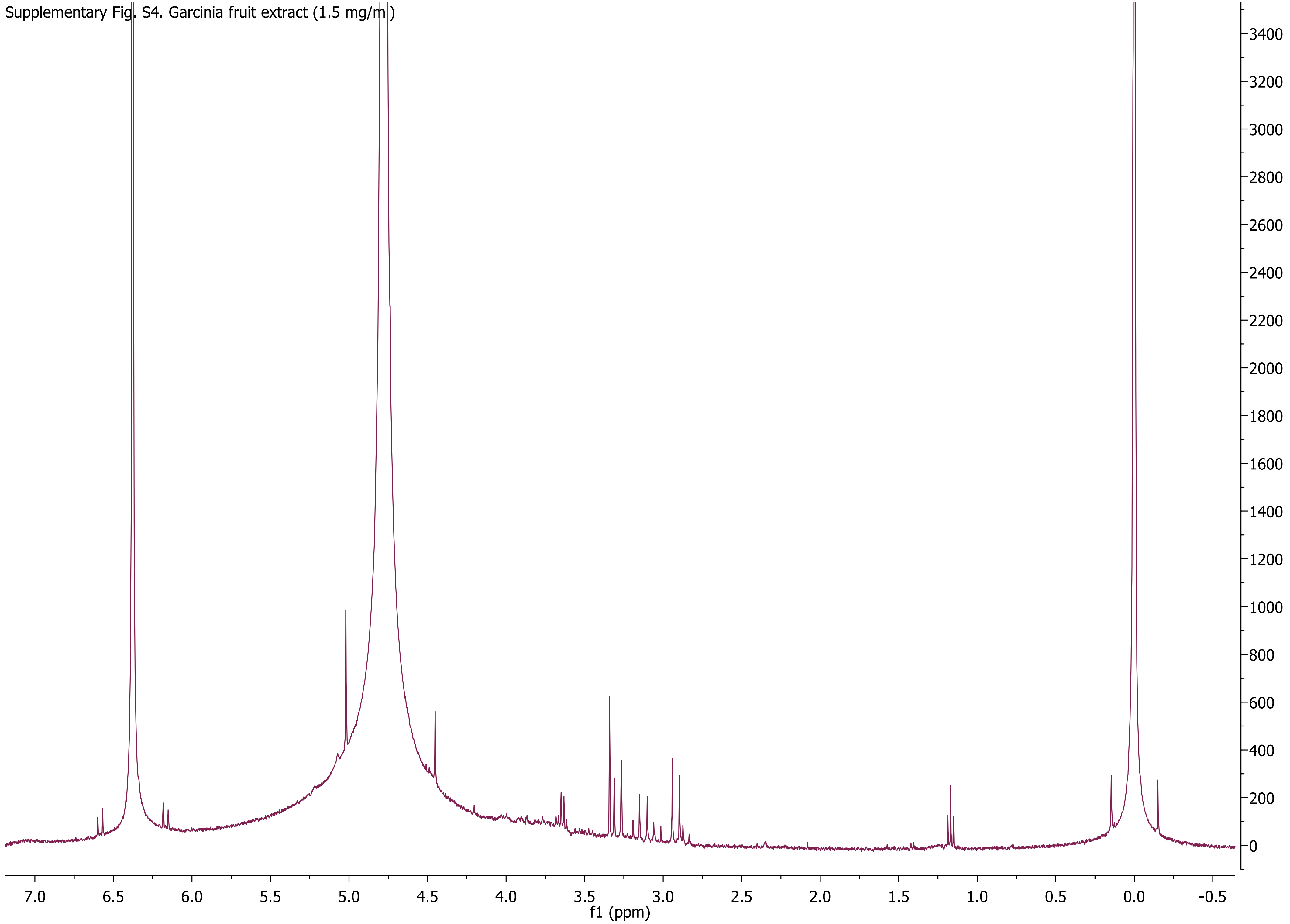

Supplementary Fig. S4. Garcinia fruit extract (0,75 mg/ml)

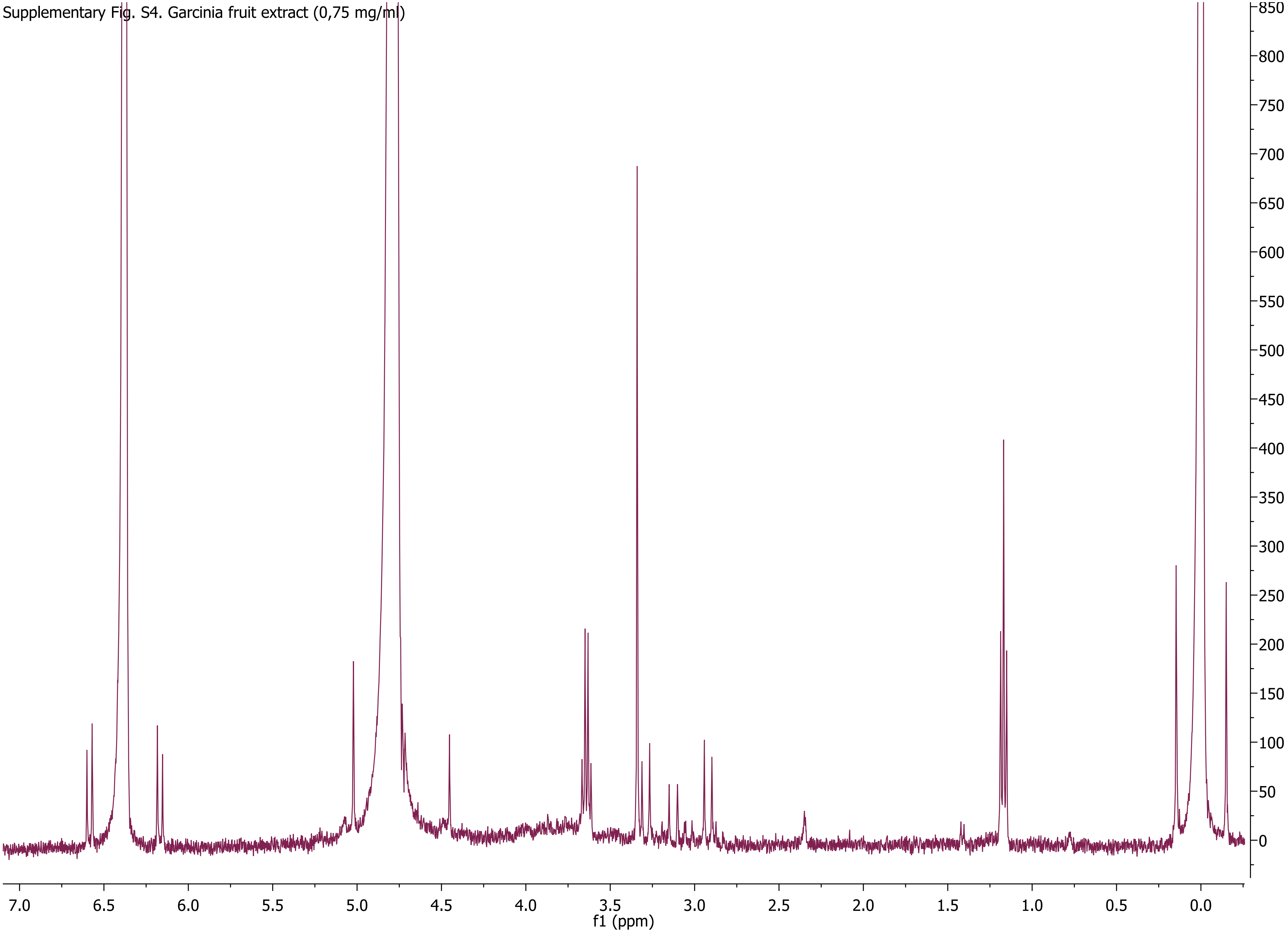

Supplementary Fig. S4. Garcinia fruit extract (0,375 mg/ml)

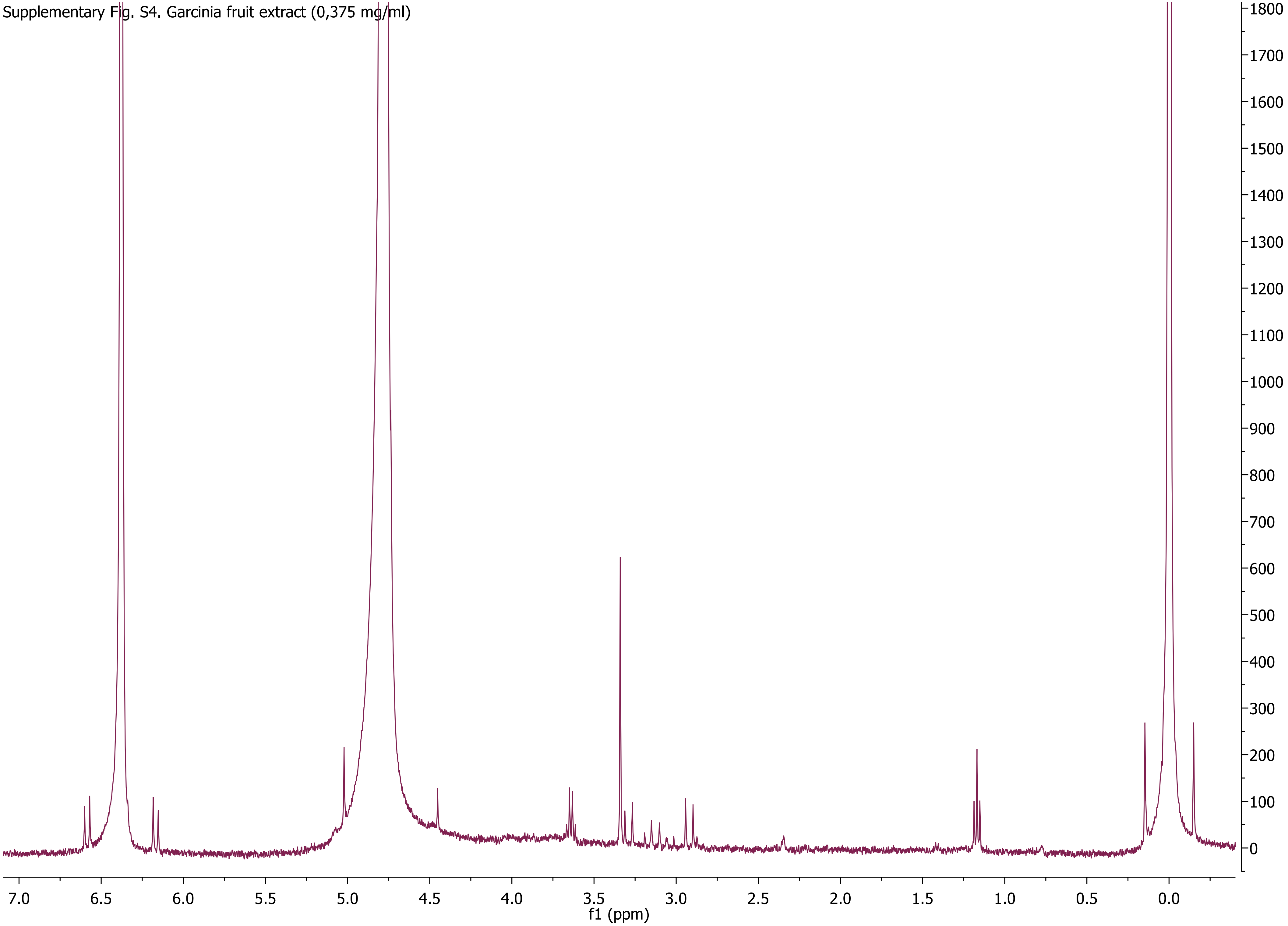

Supplementary Fig. S4. Garcinia fruit extract (0,1875 mg/ml)

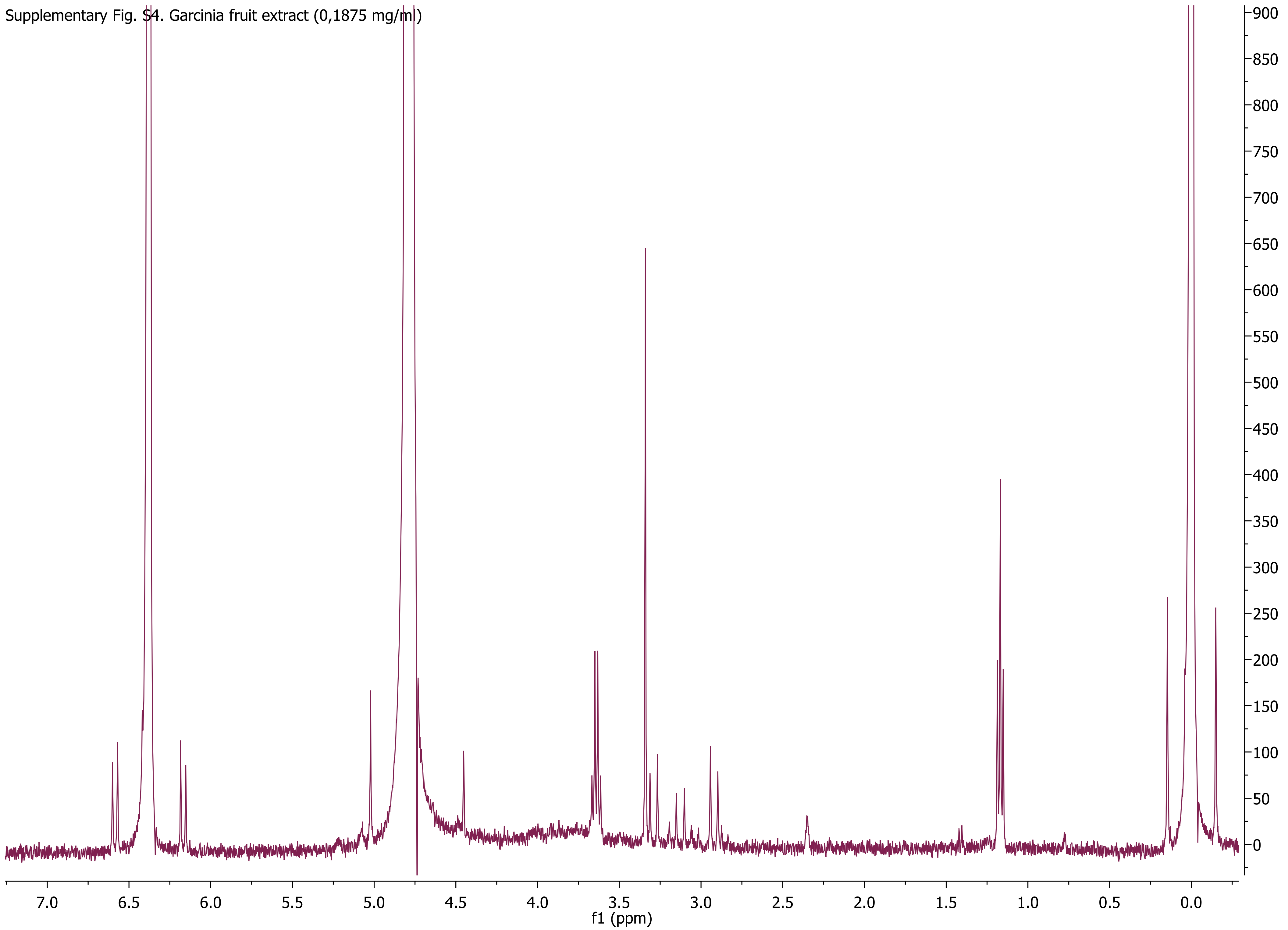

Supplementary Fig. S5. ^{1}H NMR spectrum of *Garcinia* fruits extract

Supplementary Fig. S5. Garcinia fruit (HAS388)

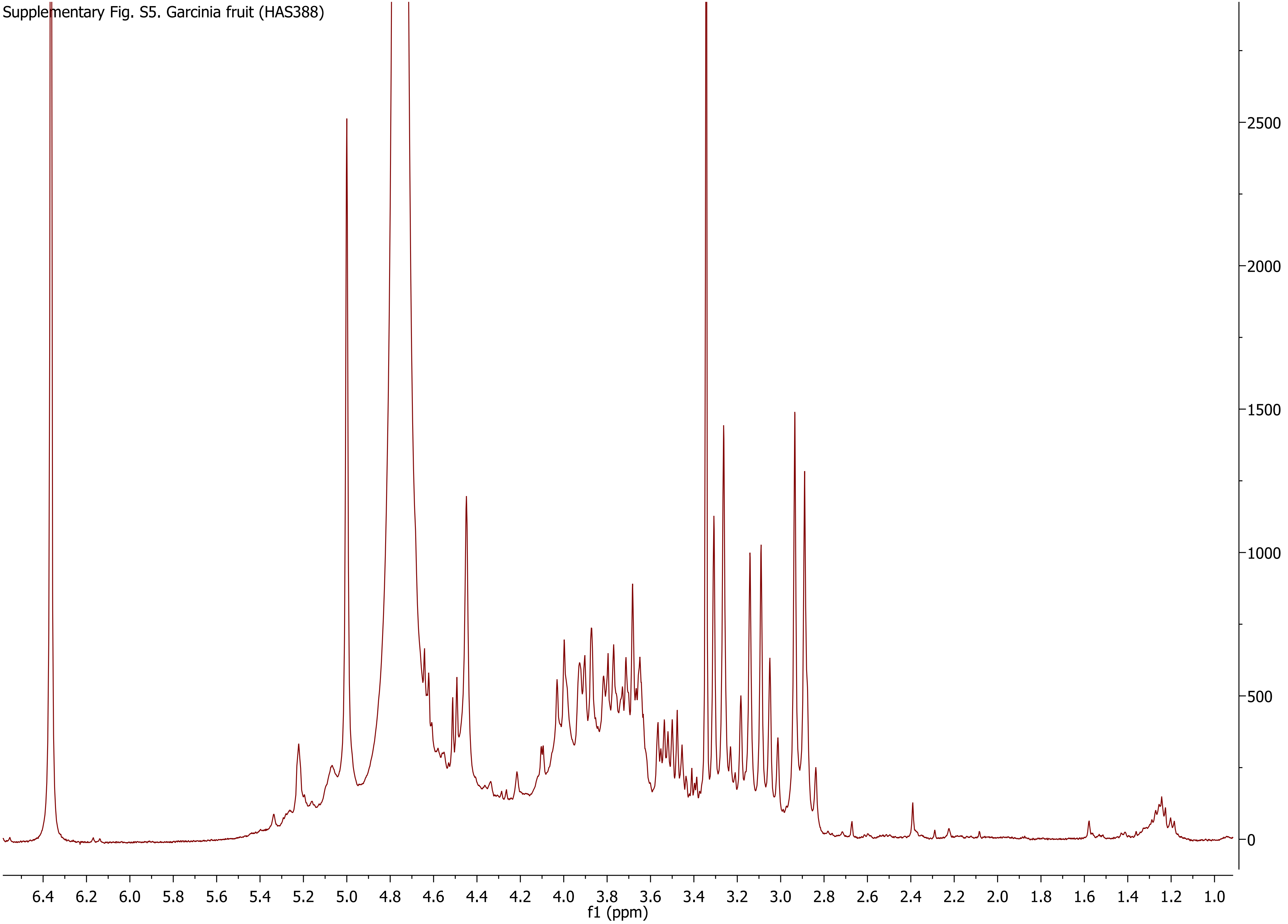

Supplementary Fig. S5. Garcinia fruit (HAS389)

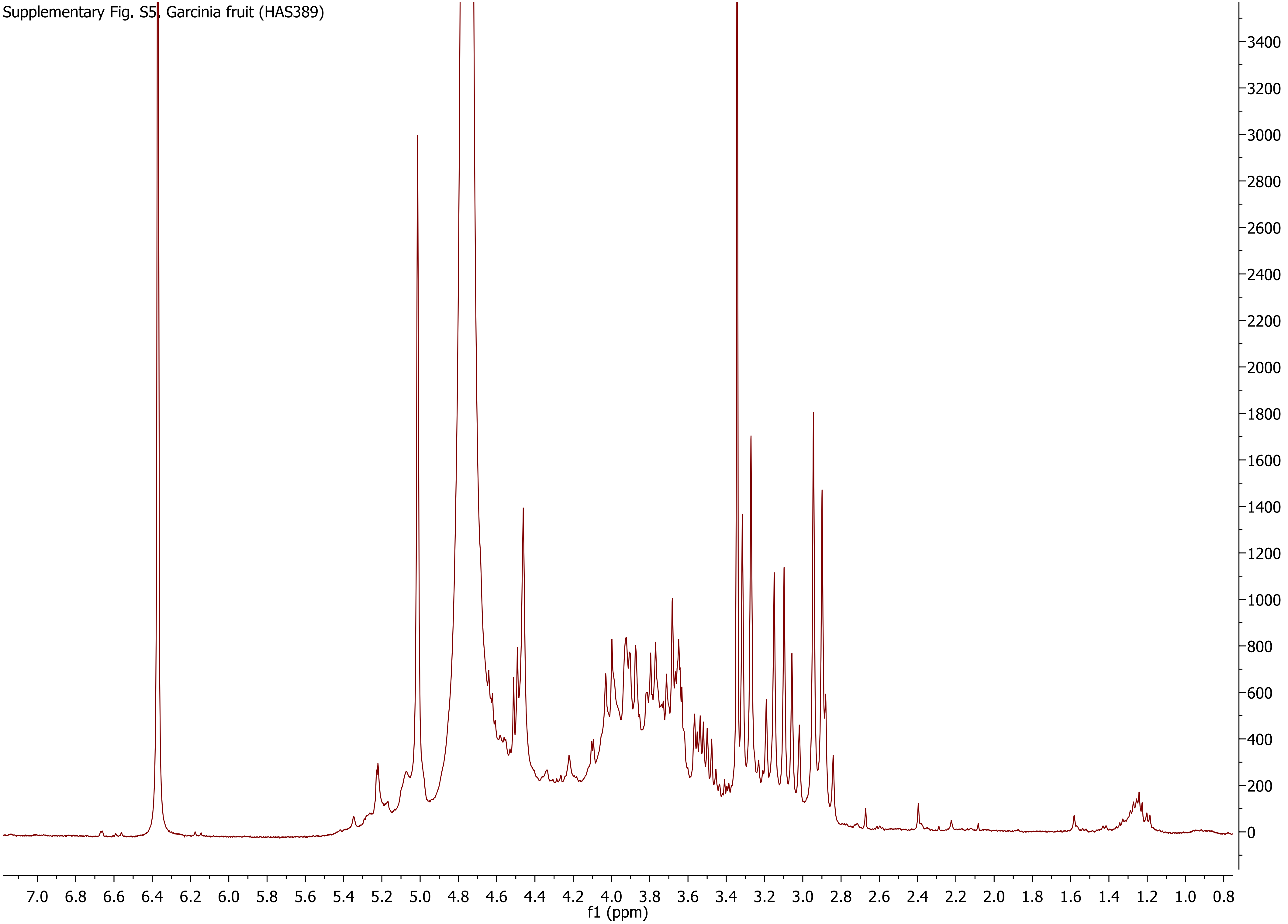

Supplementary Fig. S5. Garcinia fruit (HAS473)

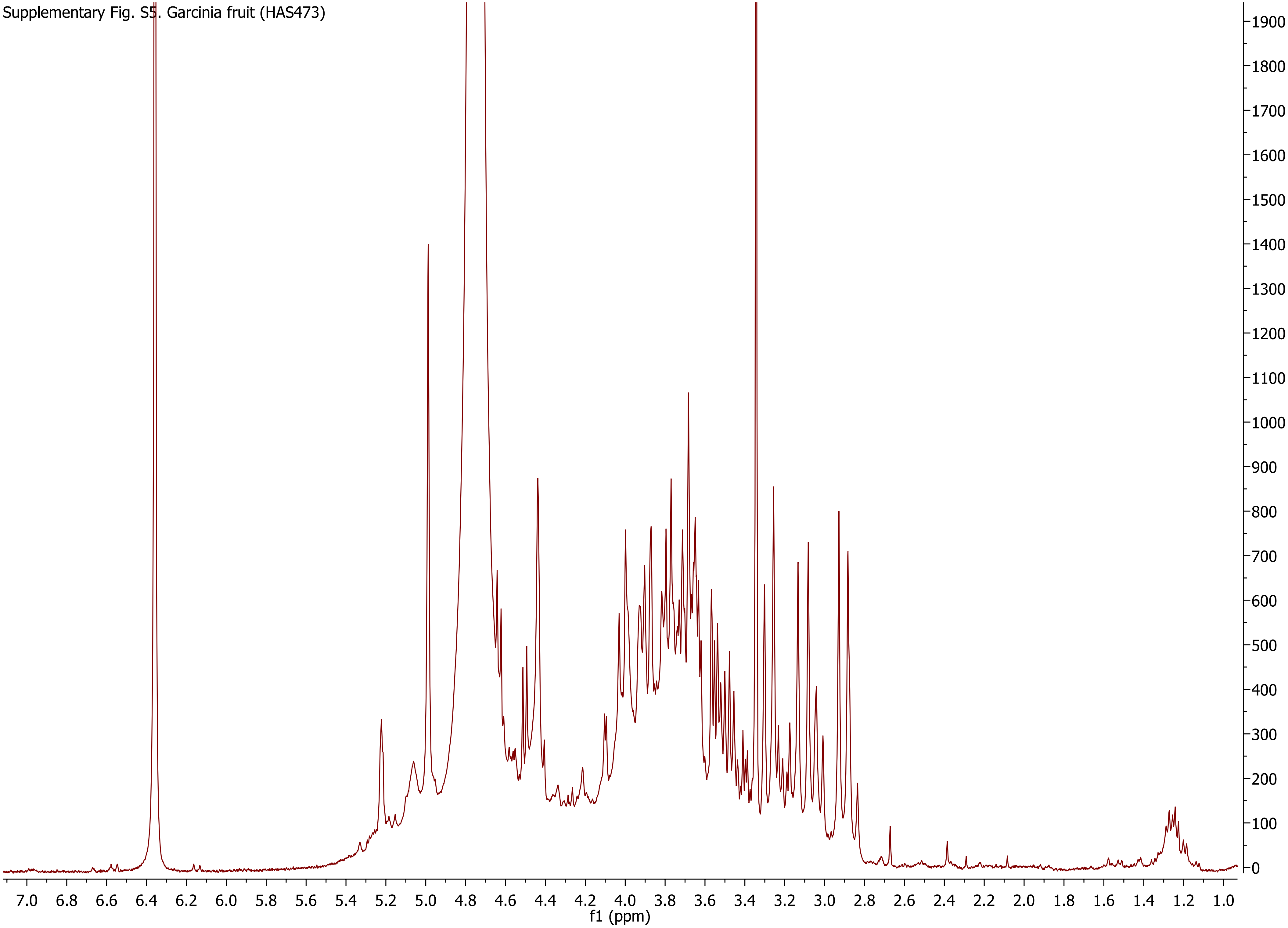

Supplementary Fig. S5. Garcinia fruit (HAS288)

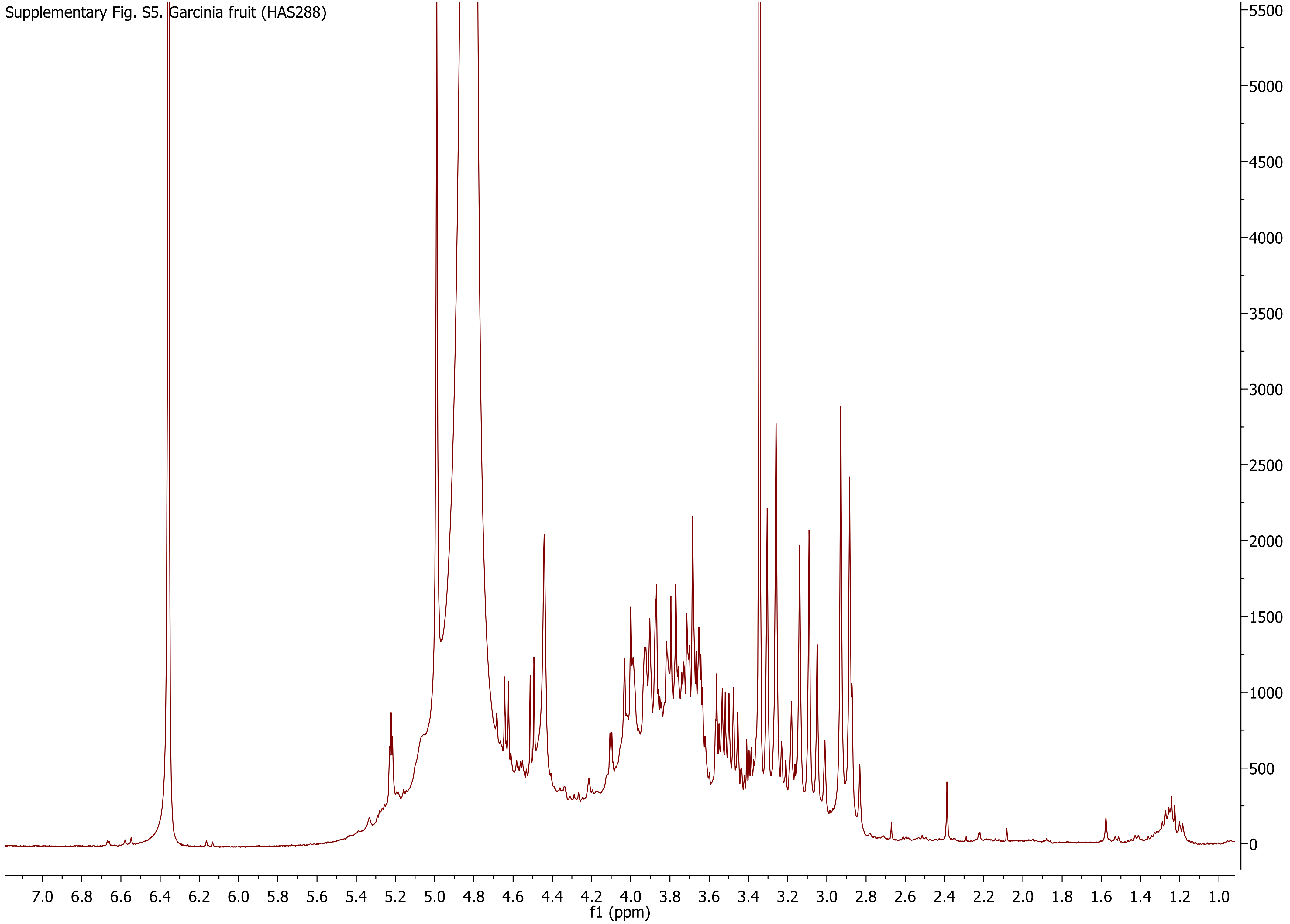

Supplementary Fig. S5. Garcinia fruit (HAS470)

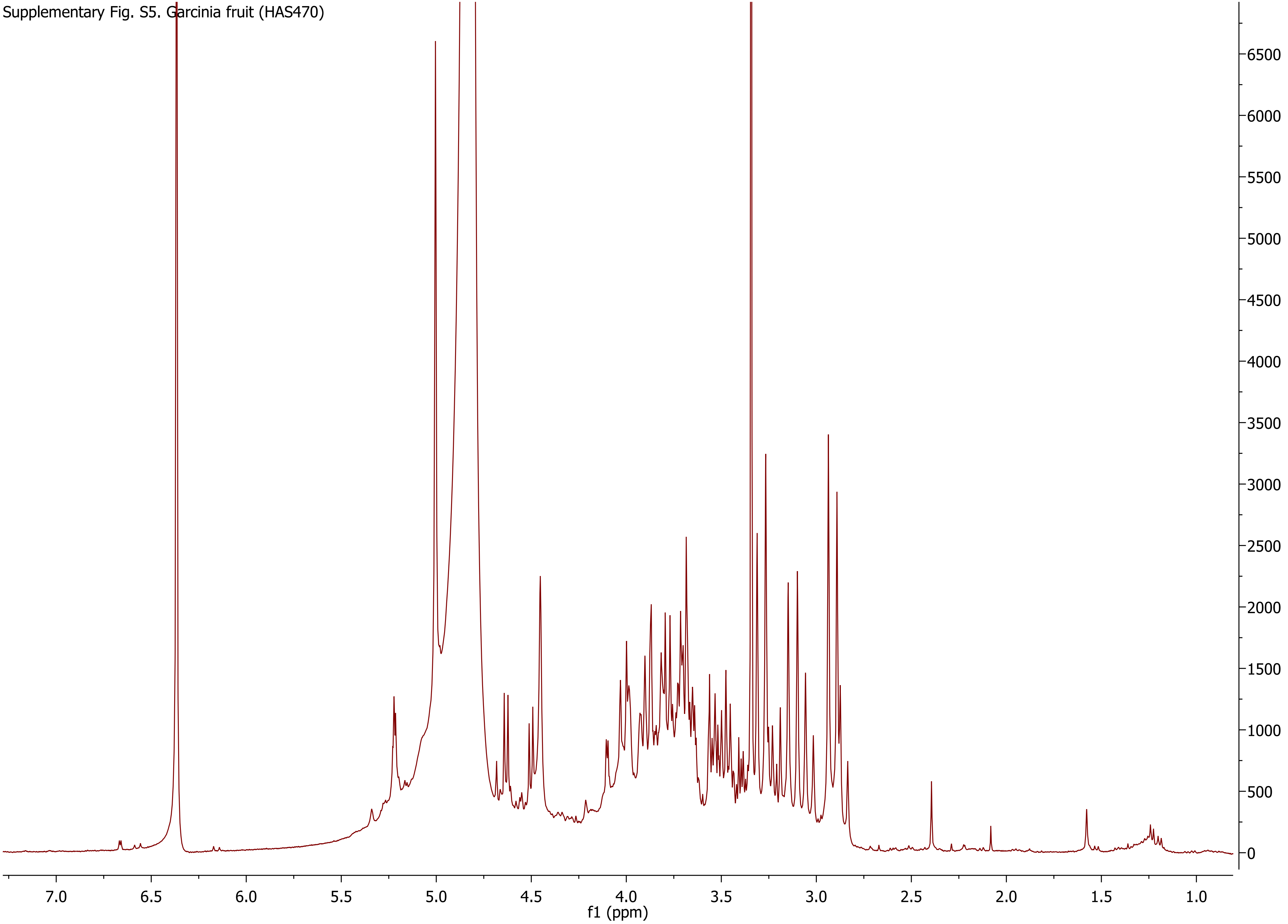

Supplementary Fig. S5. Garcinia fruit (HAS203)

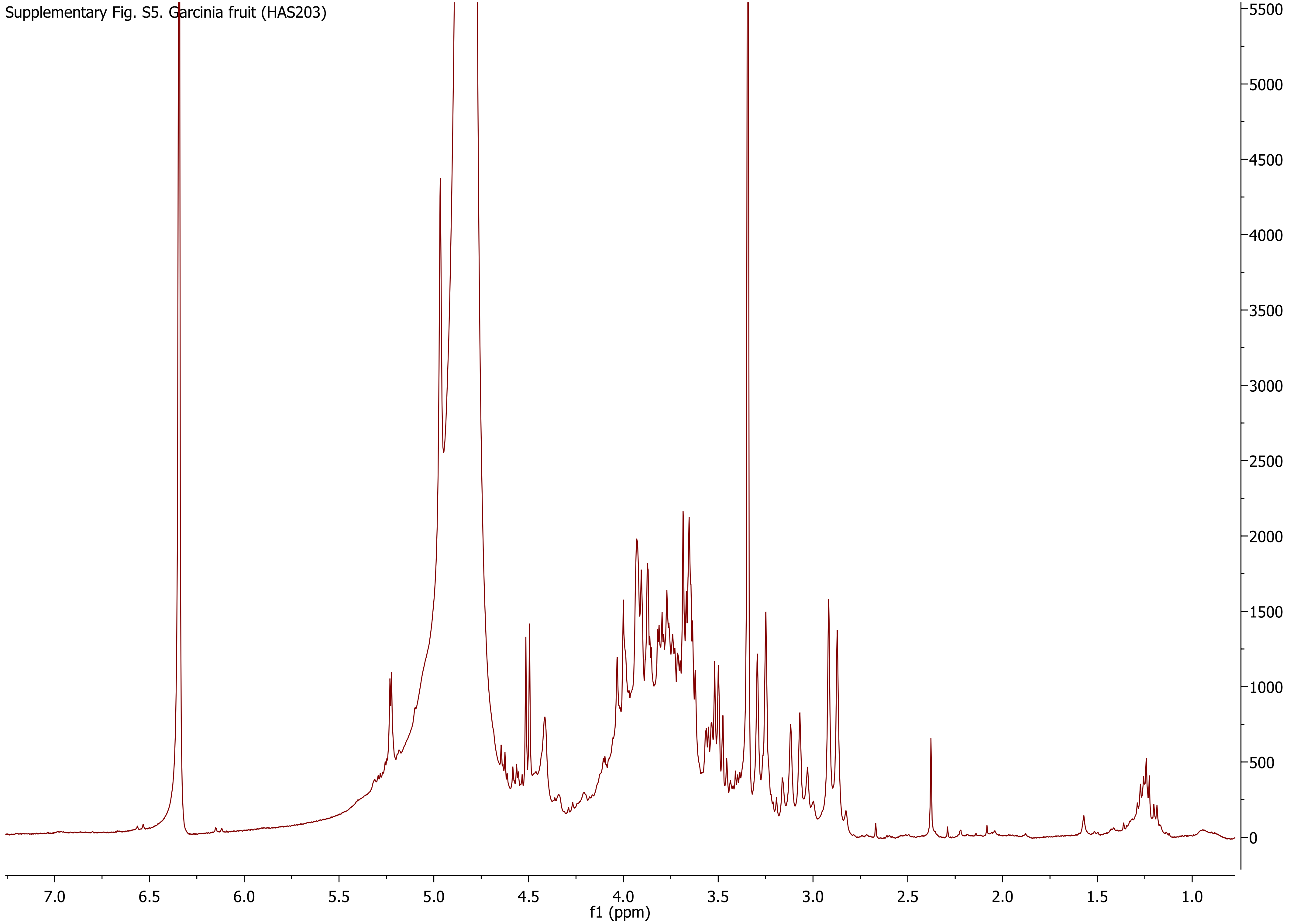

Supplementary Fig. S5. Garcinia fruit (HAS414)

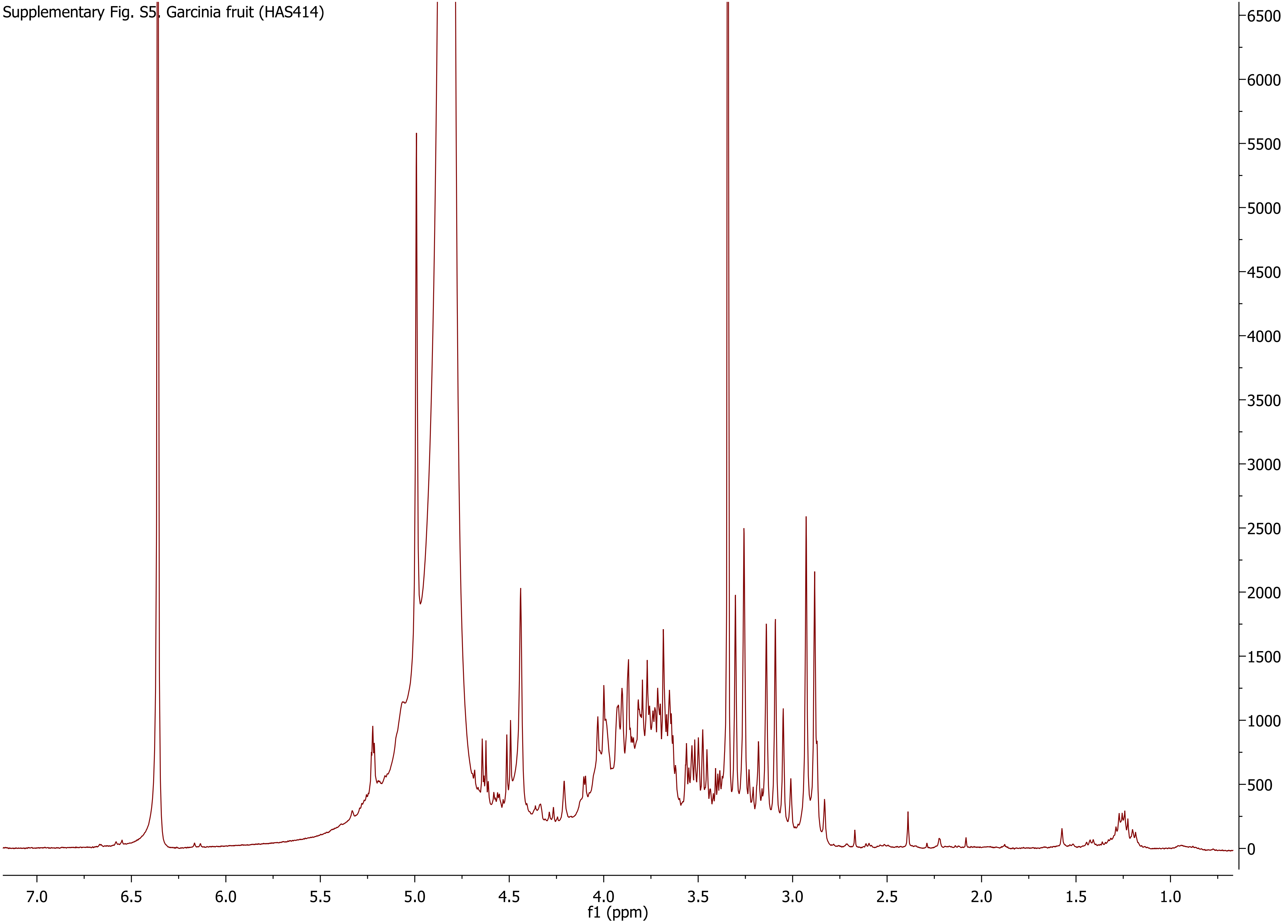

Supplementary Fig. S5. Garcinia fruit (HAS399)

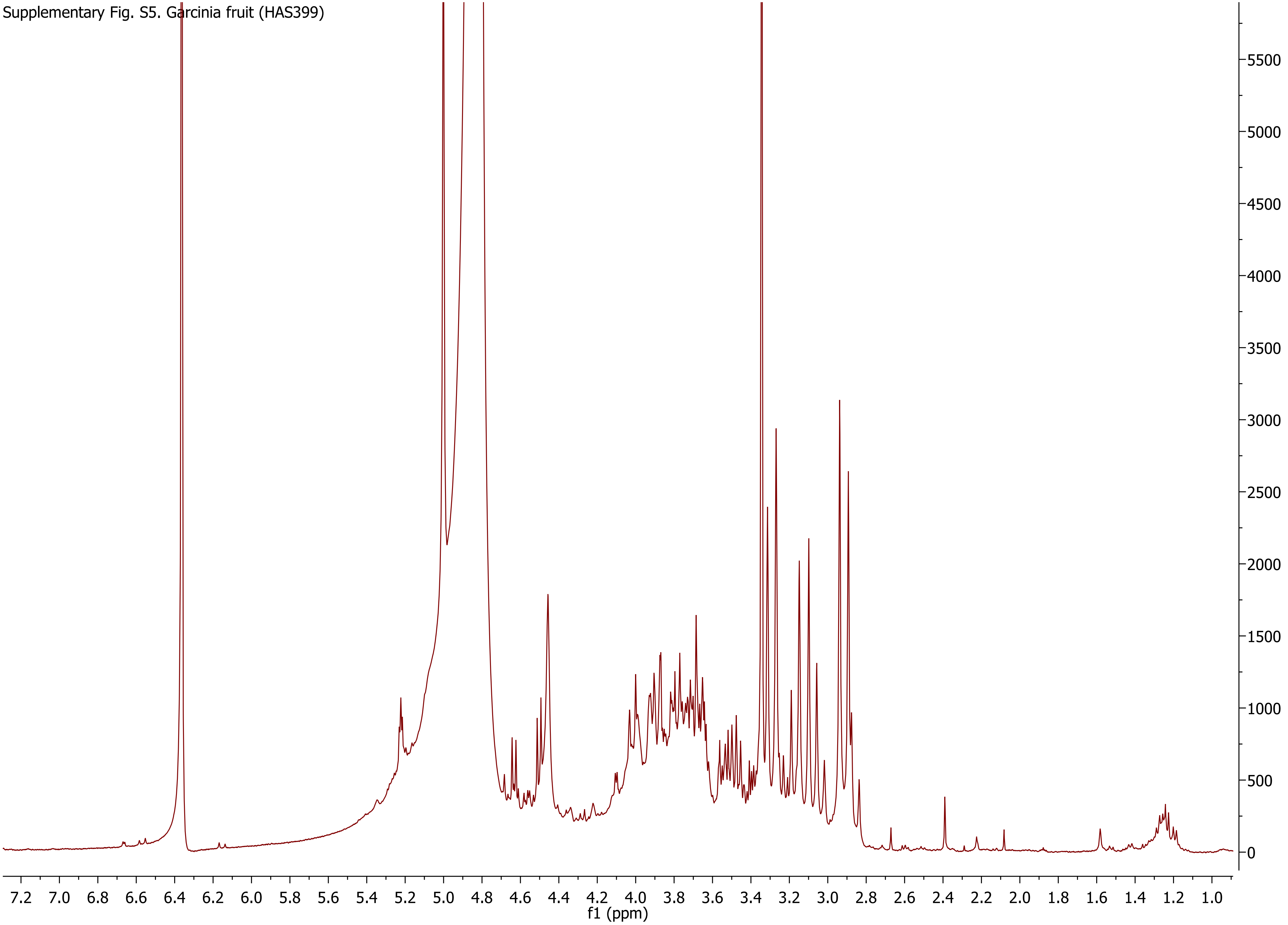

Supplementary Fig. S5. Garcinia fruit (HAS365)

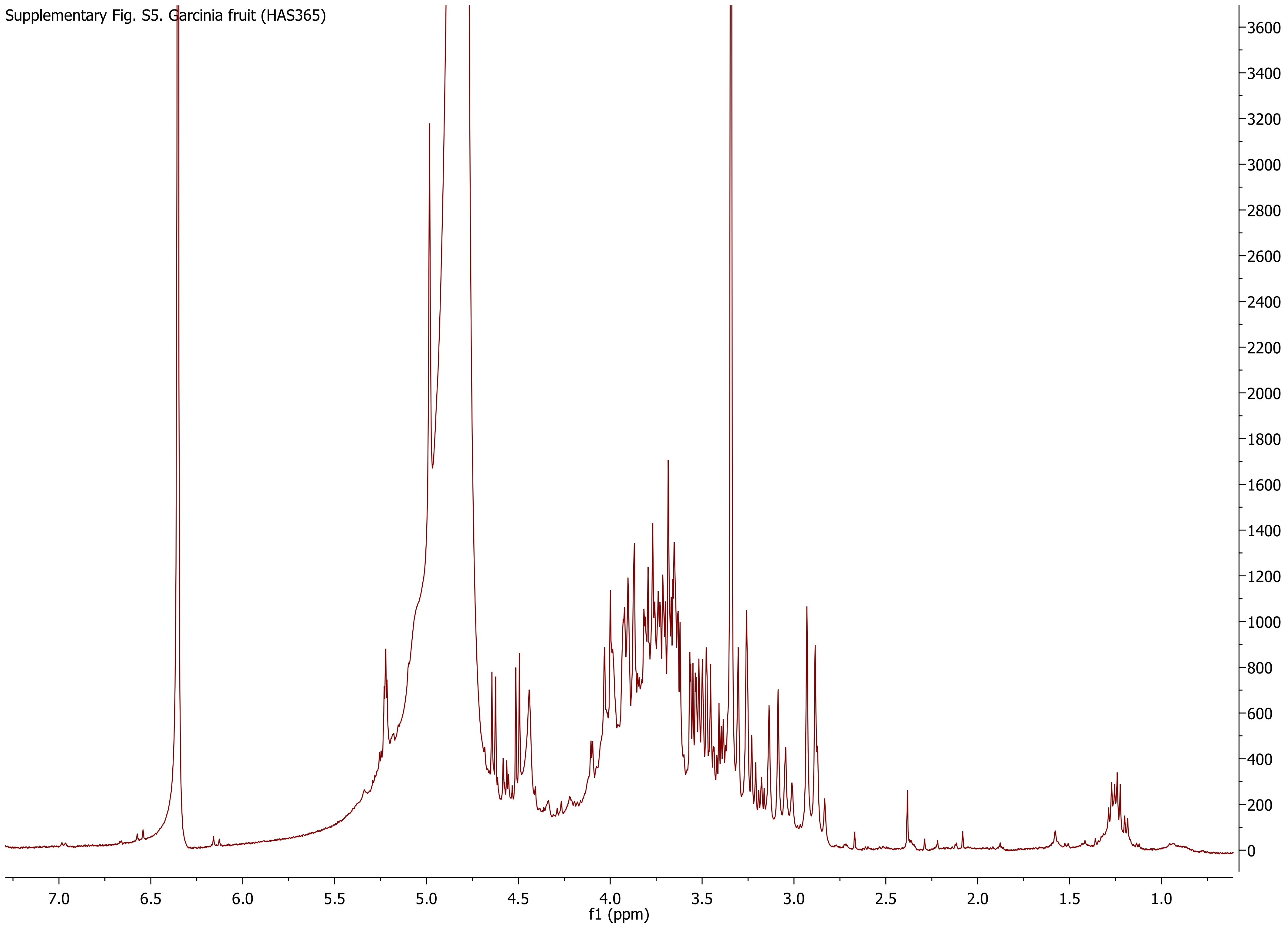

Supplementary Fig. S5. Garcinia fruit (HAS291)

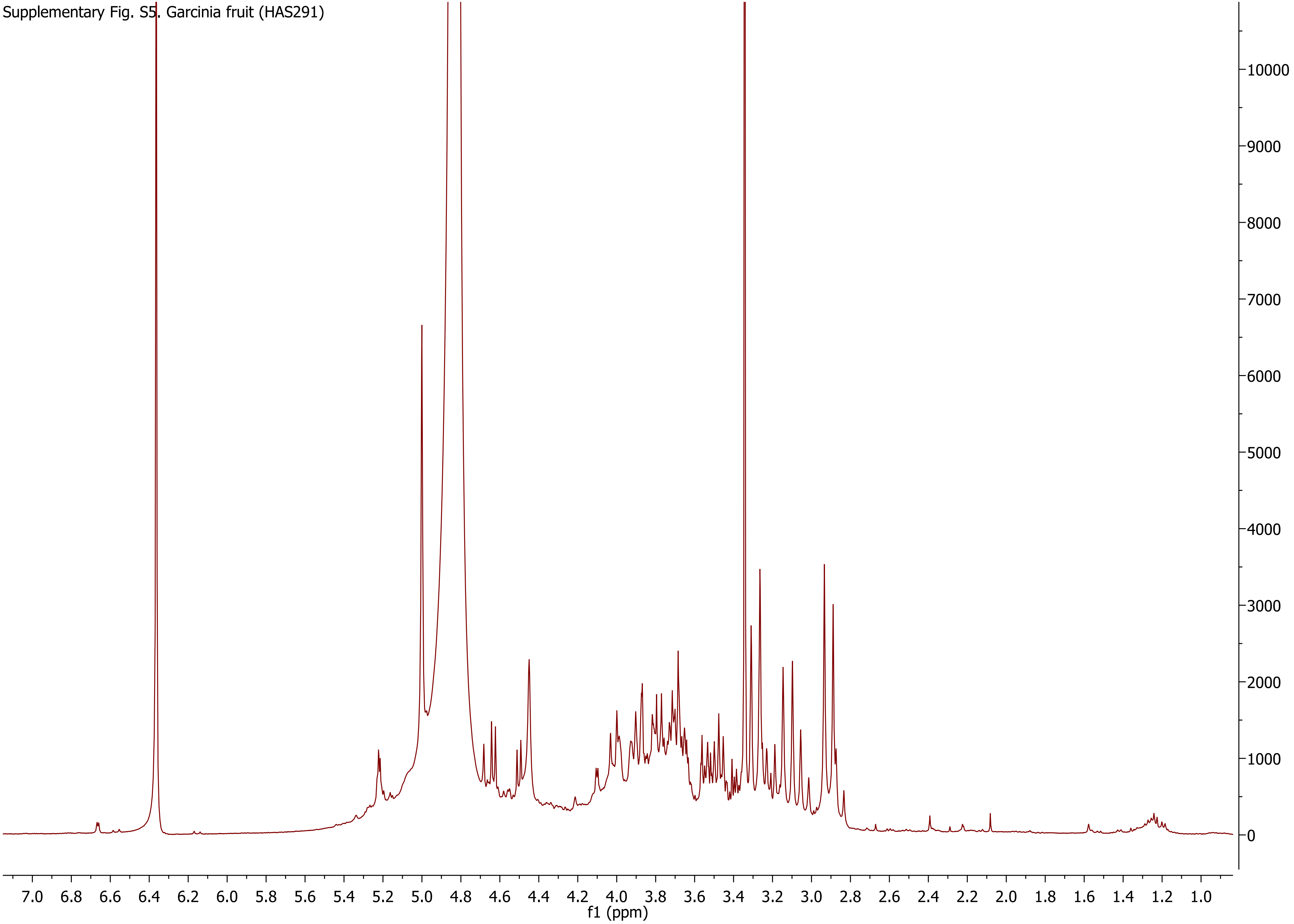

Supplementary Fig. S6. ^{1}H NMR spectrum of analyzed *Garcinia* food supplements.

Supplementary Fig. S6. Garcinia food supplement-1

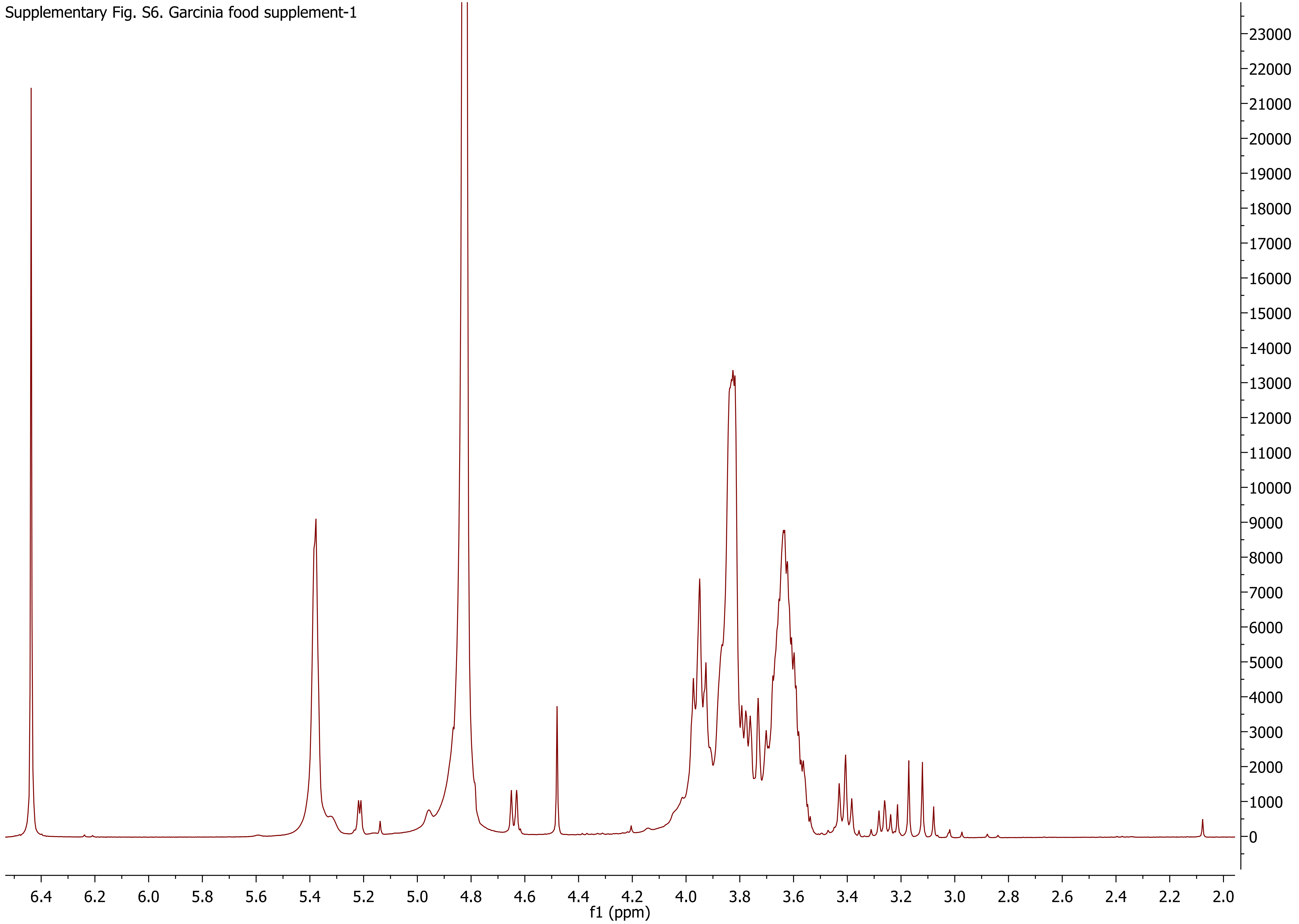

Supplementary Fig. S6. Garcinia food supplement-2

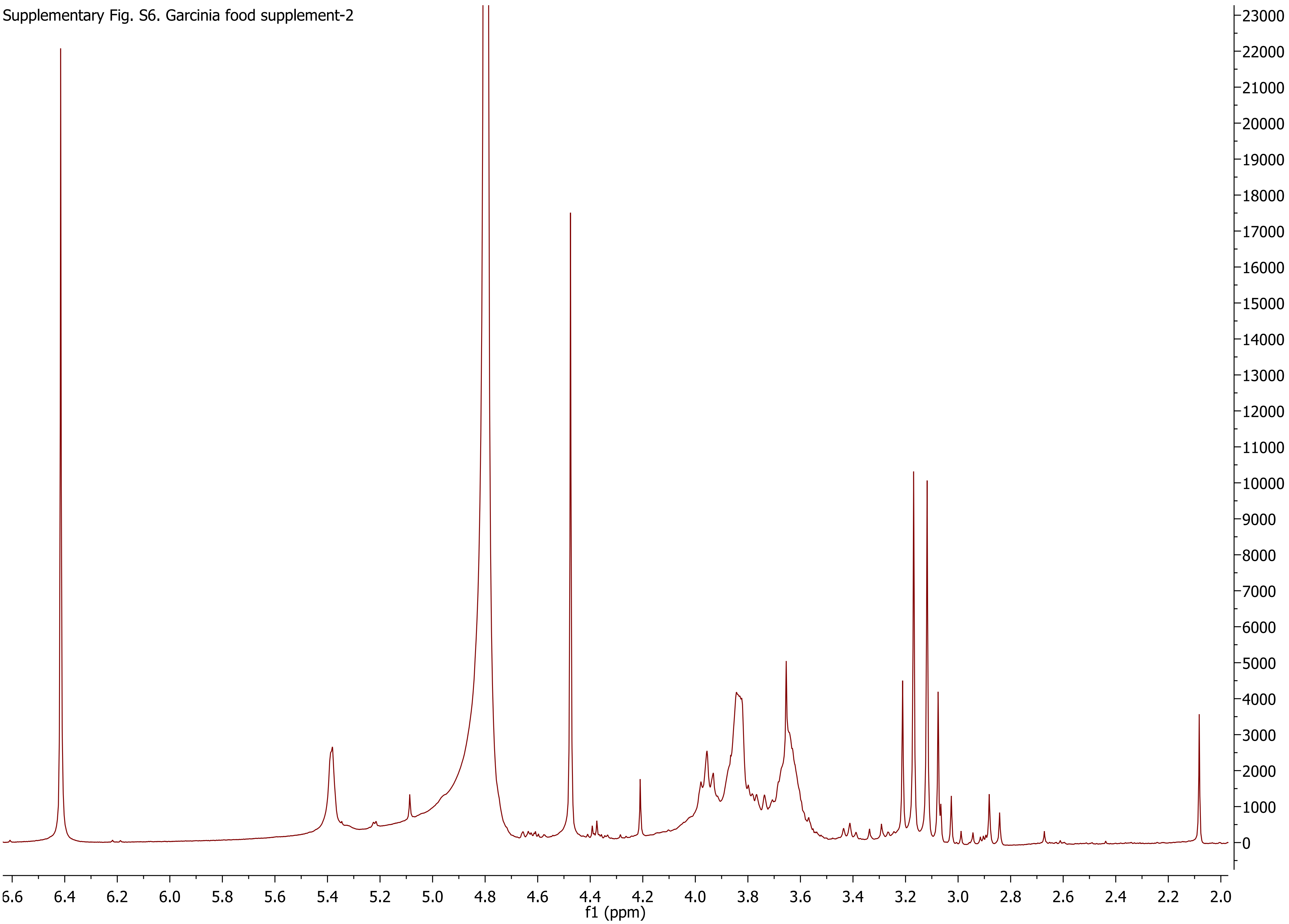

Supplementary Fig. S6. Garcinia food supplement-3

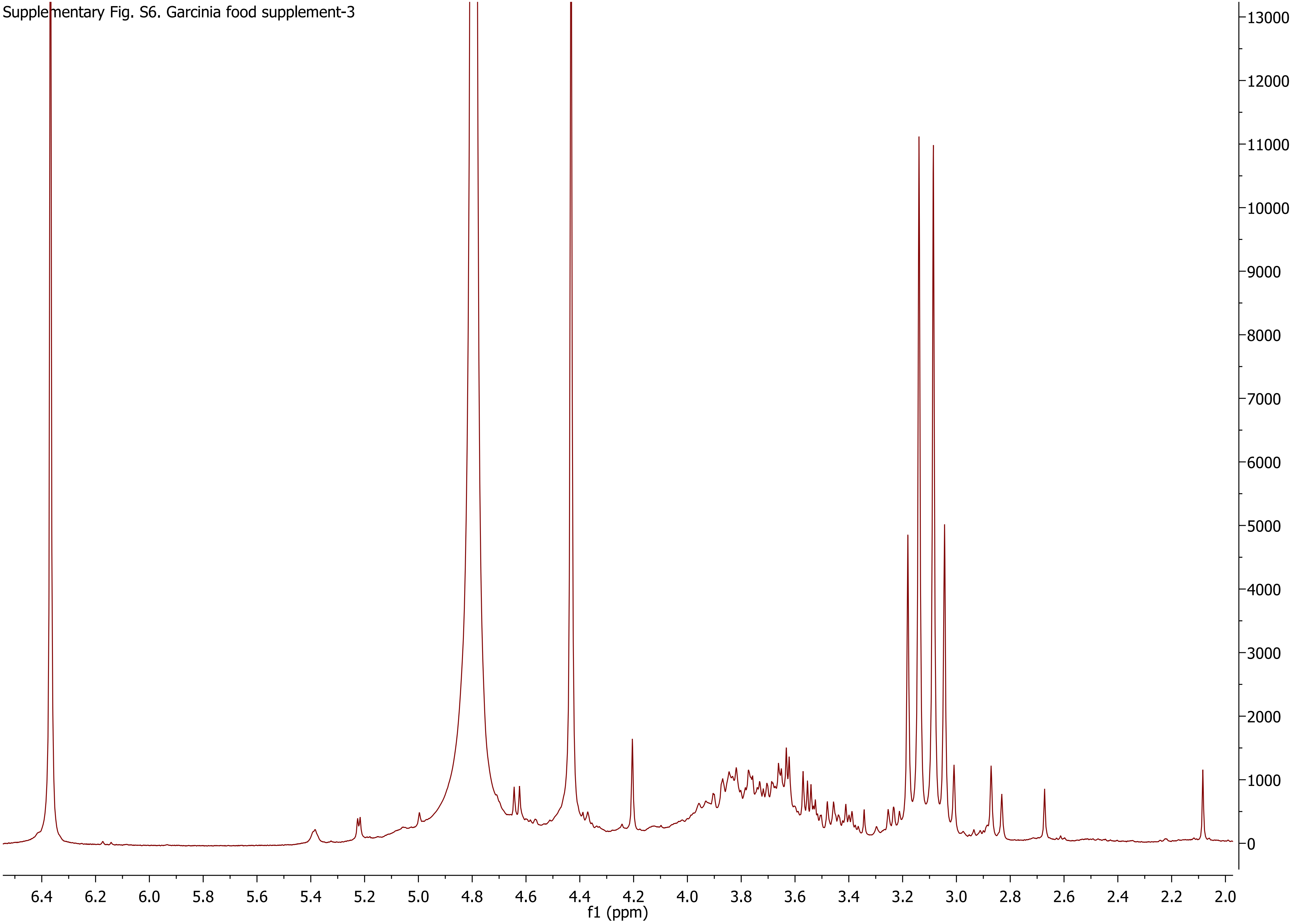

Supplementary Fig. S6. Garcinia food supplement-4

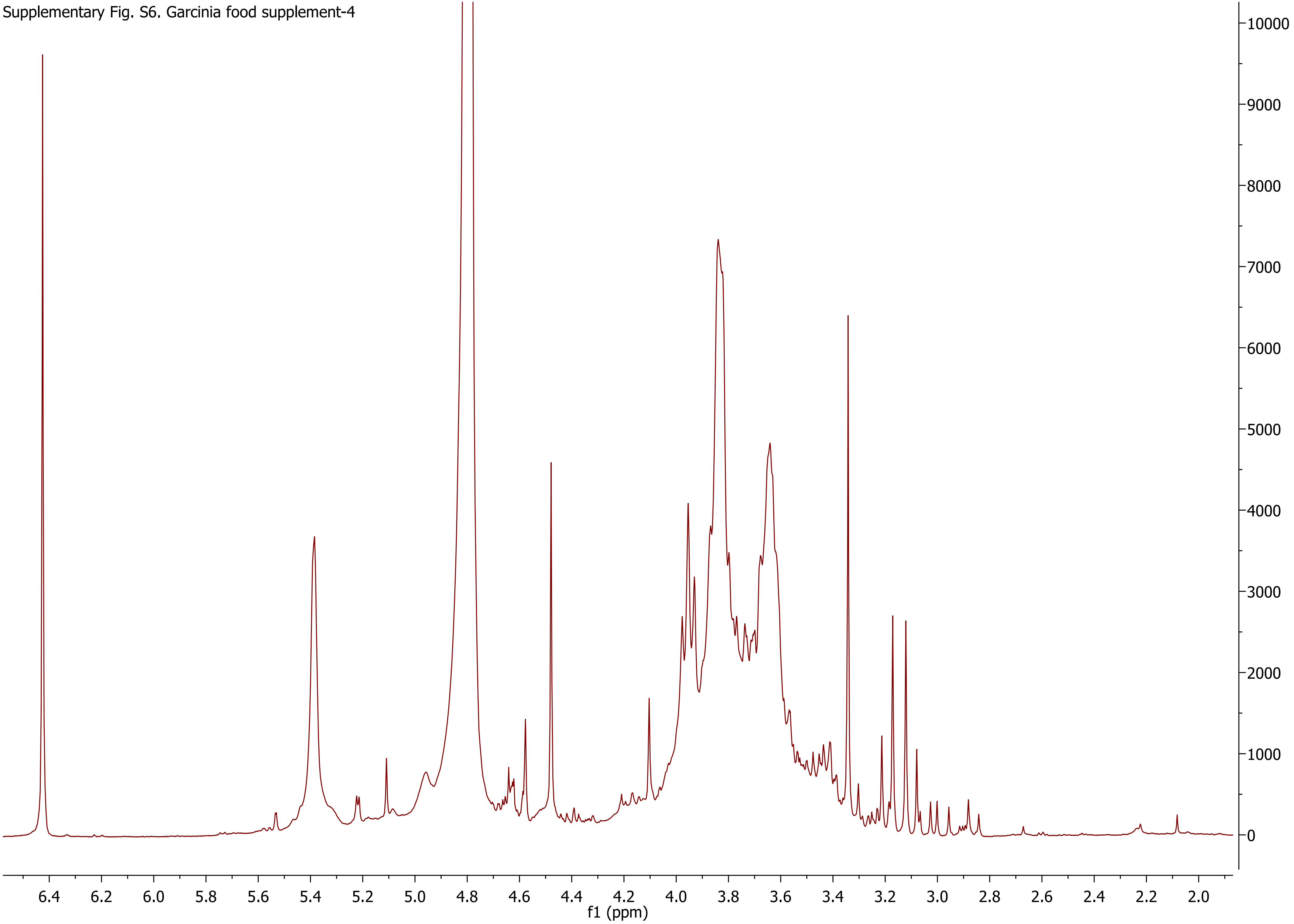

Supplementary Fig. S6. Garcinia food supplement-5

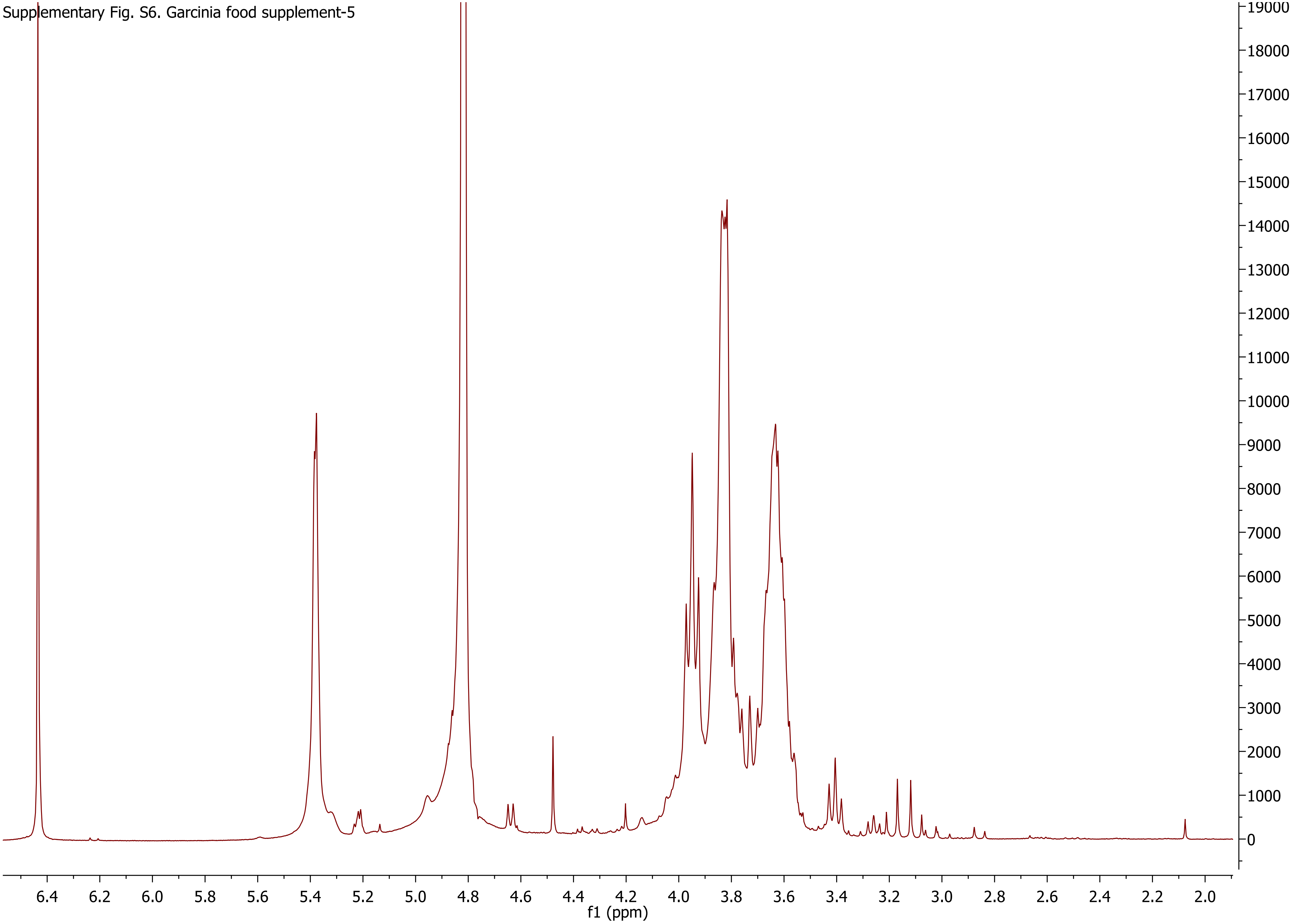

Supplementary Fig. S6. Garcinia food supplement-6

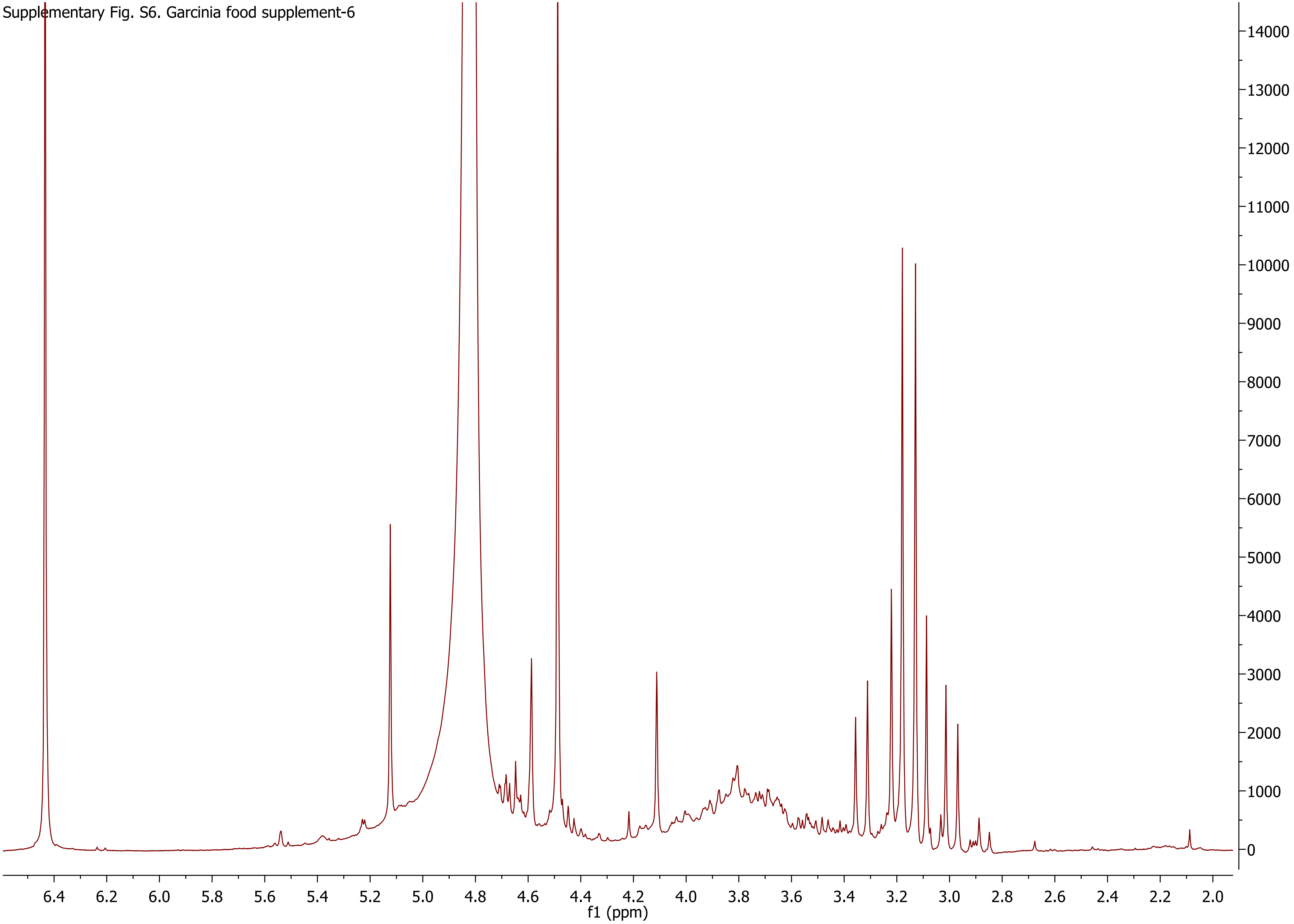

Supplementary Fig. S6. Garcinia food supplement-7

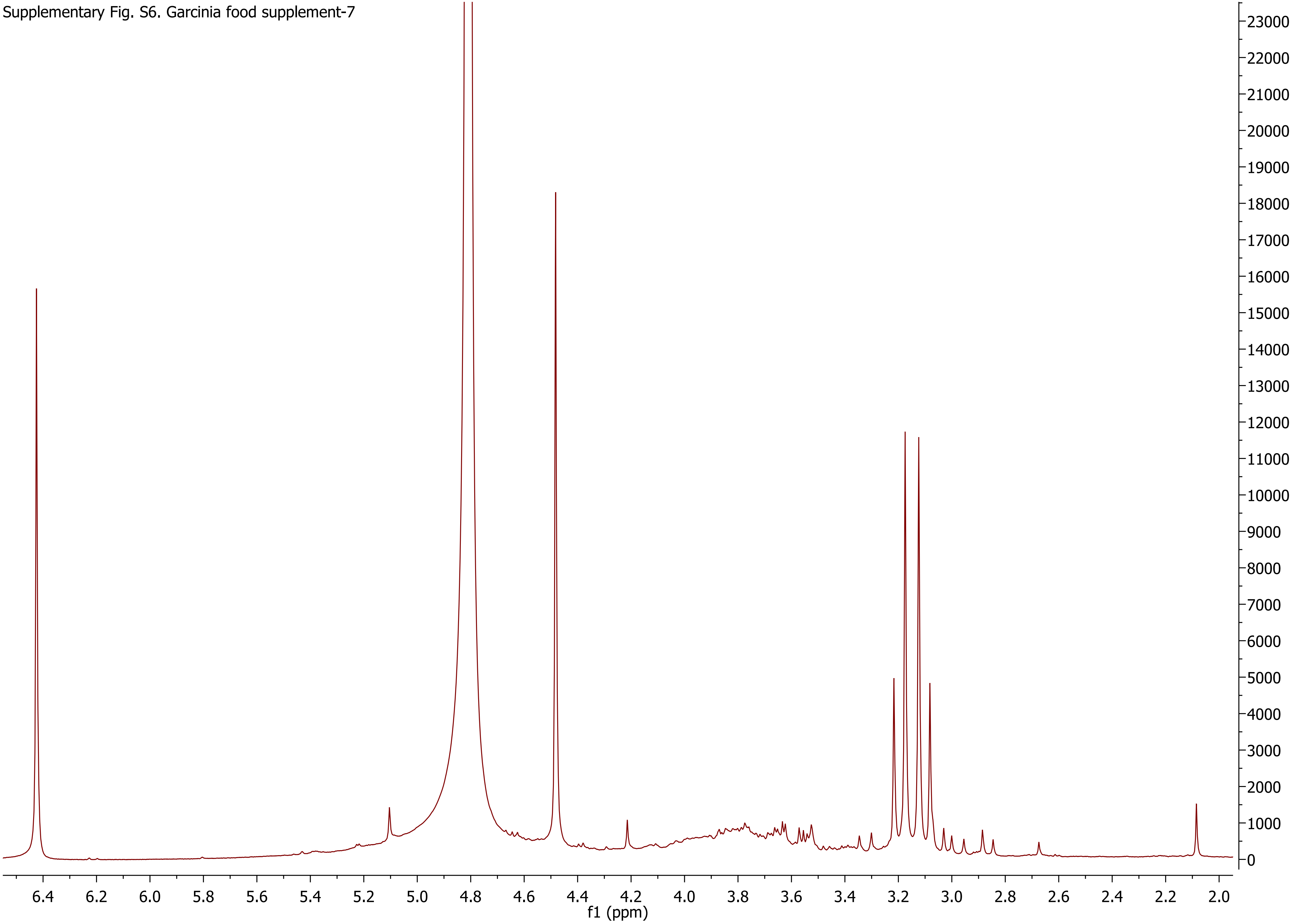

Supplementary Fig. S6. Garcinia food supplement-8

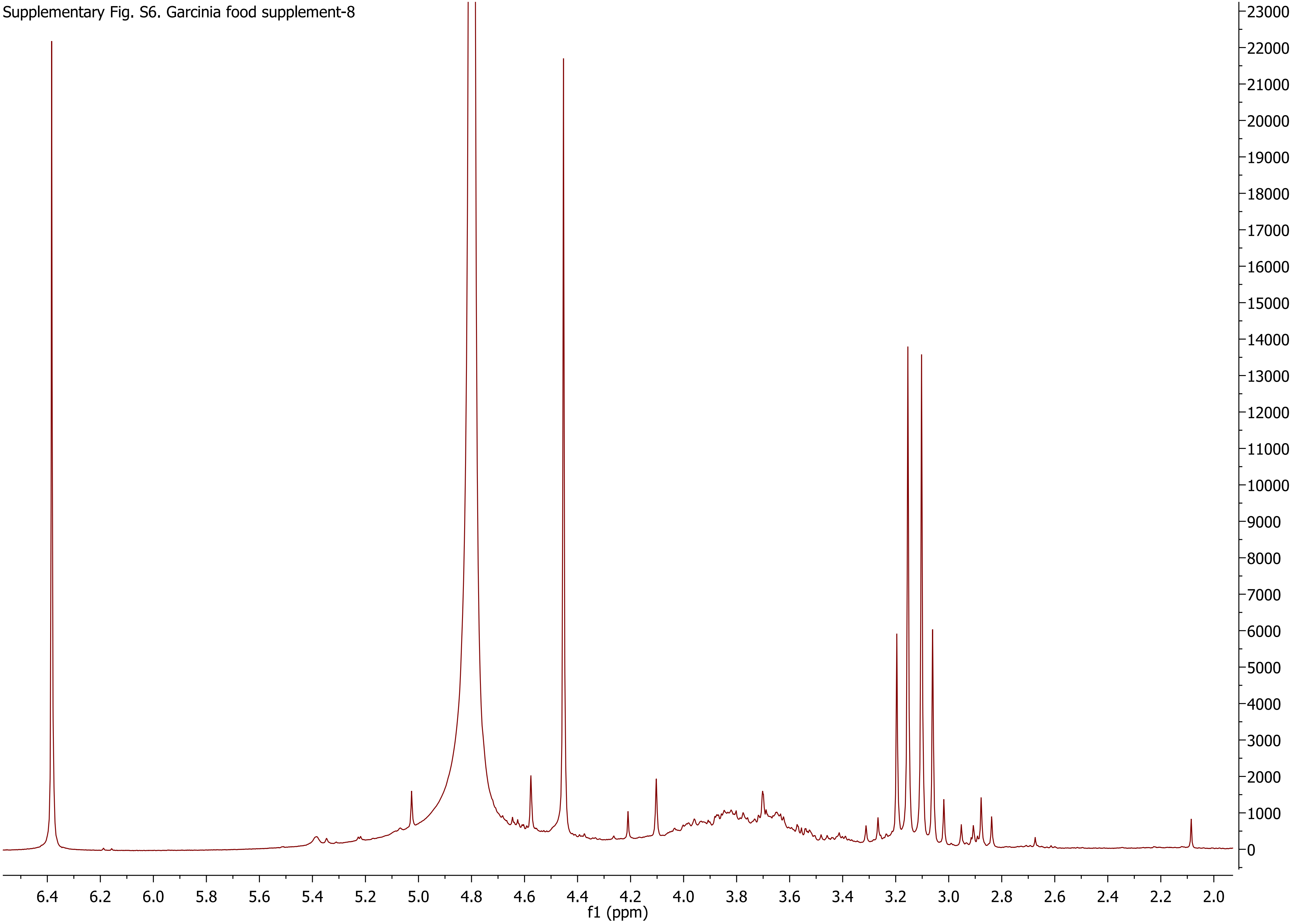

Supplementary Fig. S6. Garcinia food supplement-9

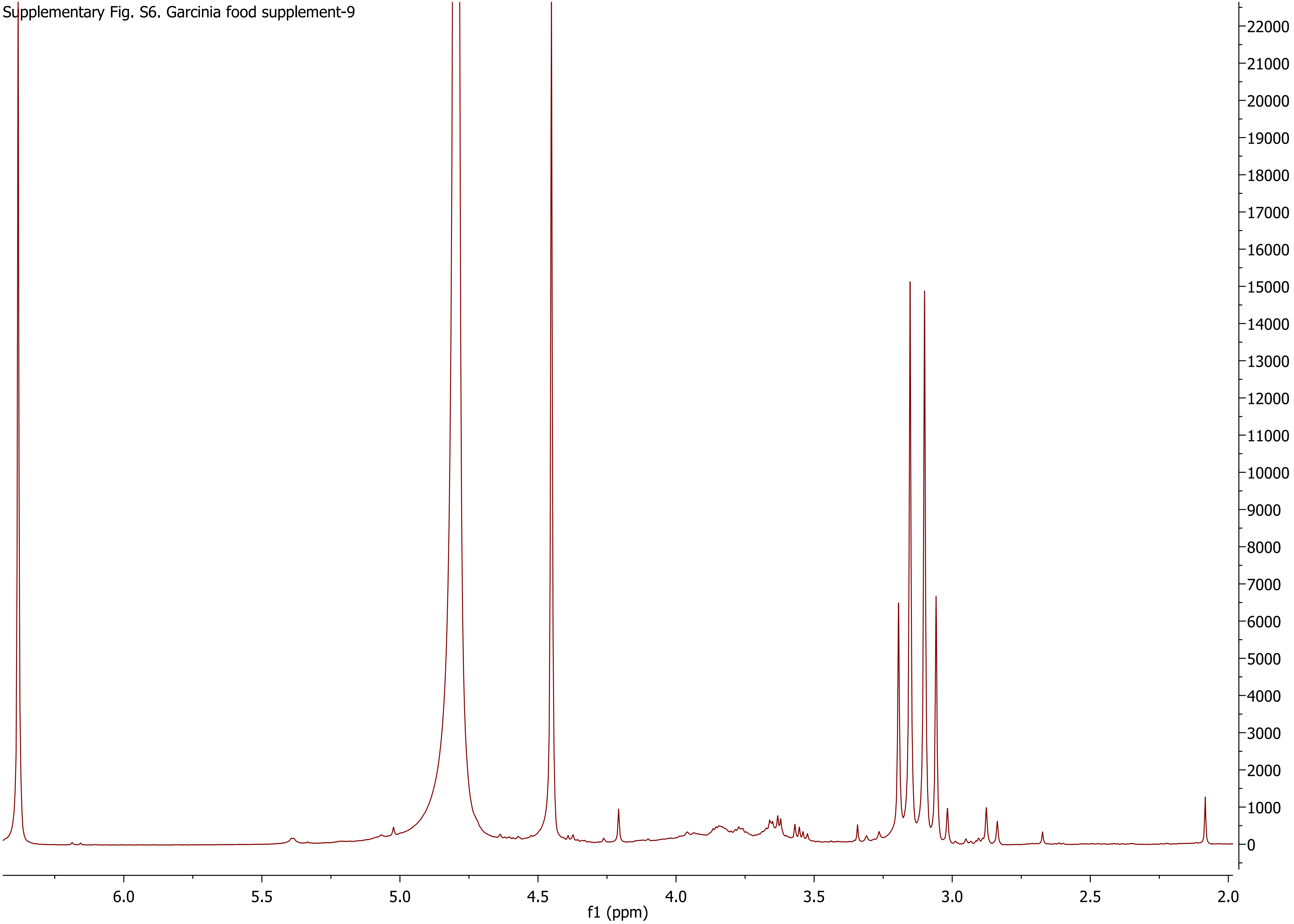

Supplementary Fig. S6. Garcinia food supplement-10

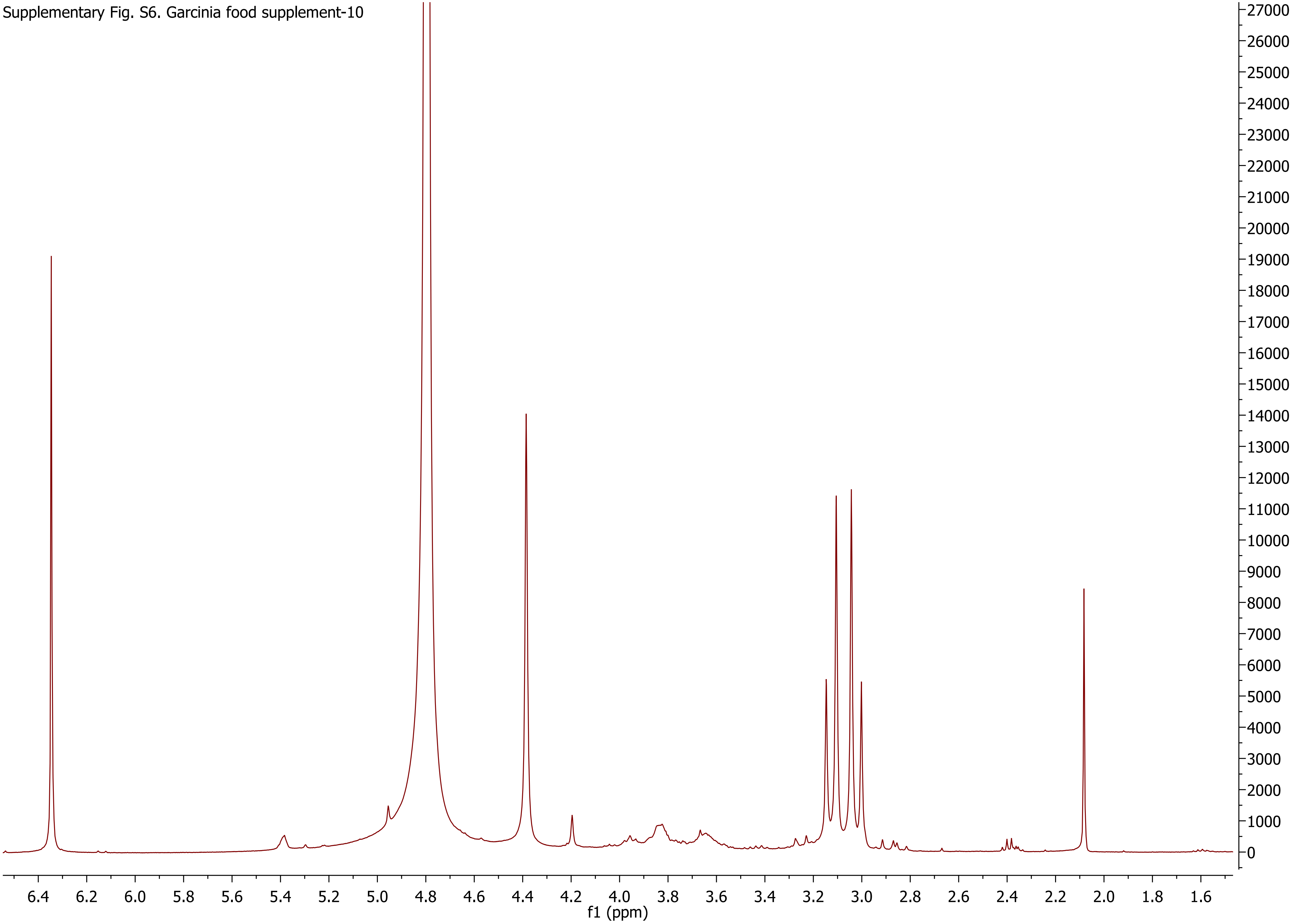

Supplement: Supplementary file 1 — Supplementary Information [file 41598_2018_28635_MOESM1_ESM.pdf]
